# Cryo-Electron Microscopy Structure and Interactions of the Human Cytomegalovirus gHgLgO Trimer with Platelet-Derived Growth Factor Receptor Alpha

**DOI:** 10.1128/mBio.02625-21

**Published:** 2021-10-26

**Authors:** Jing Liu, Adam Vanarsdall, Dong-Hua Chen, Andrea Chin, David Johnson, Theodore S. Jardetzky

**Affiliations:** a Department of Structural Biology, Stanford University grid.168010.egrid.471392.a School of Medicine, Stanford, California, USA; b Department of Molecular Microbiology & Immunology, Oregon Health & Science University, Portland, Oregon, USA; Columbia University

**Keywords:** human cytomegalovirus, trimer, PDGFRα, cryo-EM, receptor complex, HCMV, pentamer, virus entry

## Abstract

Human cytomegalovirus (HCMV) is a herpesvirus that produces disease in transplant patients and newborn children. Entry of HCMV into cells relies on gH/gL trimer (gHgLgO) and pentamer (gHgLUL128–131) complexes that bind cellular receptors. Here, we studied the structure and interactions of the HCMV trimer, formed by AD169 strain gH and gL and TR strain gO proteins, with the human platelet-derived growth factor receptor alpha (PDGFRα). Three trimer surfaces make extensive contacts with three PDGFRα N-terminal domains, causing PDGFRα to wrap around gO in a structure similar to a human hand, explaining the high-affinity interaction. gO is among the least conserved HCMV proteins, with 8 distinct genotypes. We observed high conservation of residues mediating gO-gL interactions but more extensive gO variability in the PDGFRα interface. Comparisons between our trimer structure and a previously determined structure composed of different subunit genotypes indicate that gO variability is accommodated by adjustments in the gO-PDGFRα interface. We identified two loops within gO that were disordered and apparently glycosylated, which could be deleted without disrupting PDGFRα binding. We also identified four gO residues that contact PDGFRα, which when mutated produced markedly reduced receptor binding. These residues fall within conserved contact sites of gO with PDGFRα and may represent key targets for anti-trimer neutralizing antibodies and HCMV vaccines. Finally, we observe that gO mutations distant from the gL interaction site impact trimer expression, suggesting that the intrinsic folding or stability of gO can impact the efficiency of trimer assembly.

## INTRODUCTION

Human cytomegalovirus (HCMV) is a member of the betaherpesvirus family which is widespread in the human population and responsible for a significant health burden in vulnerable groups (1–3). While HCMV infection is generally benign, infections during pregnancy can result in transmission to the fetus and cause birth defects that affect ∼0.5% of newborns (2, 4). Congenital HCMV infections can damage the nervous system of the developing fetus and account for ∼25% of children with sensorineural hearing loss in the United States (3). In addition, infections in immunocompromised transplant recipients affect up to 15 to 30% of high-risk solid organ transplants and can result in acute and chronic graft rejection. While progress has been made in controlling the impact of HCMV infections, there remains a significant need to develop new therapeutics or vaccines (5, 6).

HCMV targets a wide range of organs and tissues, infecting a variety of cells, including epithelial cells, endothelial cells, glial cells, fibroblasts, and monocytes-macrophages (7, 8). The broad range of cell types that can be infected by the virus is driven by a set of viral glycoproteins that recognize host receptors to guide and trigger virus entry. Similar to other herpesviruses, HCMV uses a common core set of viral proteins, gH, gL, and gB, as part of its entry machinery (9, 10). The gB glycoprotein is a trimeric class III fusion protein, which is activated to drive viral and cell membrane fusion after host receptor engagement (10, 11). The gHgL proteins form a core heterodimer, which associates with additional HCMV glycoproteins to form distinct higher-order complexes important to virus entry (7, 9). The association of gHgL with UL128, UL130, and UL131 creates a pentamer structure which binds to neuropilin-2 receptors on host cells (12, 13). gHgL also forms another mutually exclusive complex with the gO protein (14), creating a trimer that engages platelet-derived growth factor receptor alpha (PDGFRα) on fibroblasts (15–18). HCMV gO sequences are among the most diverse, falling into 8 different genotypes (gO1a, gO1b, gO1c, gO2a, gI2b, gO3, gO4, and gO5) that influence viral spread and antibody neutralization (19–21). By comparison, gH sequences fall within two major genotypes (gH1/gH2). The HCMV trimer is essential for entry into all cell types (22), while the pentamer enables HCMV entry into epithelial cells, endothelial cells, and monocyte-macrophages (23, 24). gHgL has also been observed to form complexes with the prefusion gB protein in biochemical and electron microscopy studies, but the functional role of this complex remains to be established (25, 26). Through their respective receptor interactions, the gHgL pentamer and trimer complexes establish the breadth of different cell types that HCMV can infect and act as regulators of gB fusogenic activity.

The pentamer and trimer proteins are essential components of the virion and the virus entry machinery, making them key targets of the neutralizing-antibody response (2, 6, 27–29). Studies of the structure, immunogenicity, and antigenicity of the HCMV pentamer complex have indicated that it is recognized by highly potent neutralizing antibodies, and the pentamer is a central focus for current vaccine development efforts (6, 27). However, the trimer also gives rise to neutralizing antibodies in human sera from transplant recipients and pregnant mothers, and antibodies to both gHgL complexes synergize to provide protection against infection (2, 30, 31). Given that the trimer plays an essential role in infection of all cell types, a better understanding of its structure, strain variability, and interactions with host receptors will provide a foundation for new approaches to developing HCMV vaccines or therapeutics and aid mechanistic studies of the viral entry process.

Here, we expressed the HCMV trimer derived from the AD169 and TR gO strains (genotypes gH1 and gO1b) and determined its structure in complex with the human PDGFRα using cryo-electron microscopy (cryo-EM). The structure reveals how gO assembles onto the gL subunit of the complex and how the three N-terminal domains of PDGFRα wrap around the globular gO domain. The gO protein contains two highly glycosylated loops oriented away from the receptor-binding surfaces, which can be deleted without disrupting the ability to form stable receptor complexes. The PDGFRα adopts a conformation distinct from that observed for PDGFRα bound to PDGF (32) but uses a substantially overlapping set of surface residues to engage the trimer. The high affinity of the trimer-PDGFRα interaction is determined by extensive interactions over four distinct contact sites, one involving gH that is less extensive and three distinct sites in gO. Two of the sites in gO that contact PDGFRα are also anticipated to contact PDGF. Site-directed mutations throughout the gO interface map a subset of key interactions with the receptor, although the large contact area in the trimer-PDGFRα complex makes it resistant to the majority of single point mutations. Comparisons to a recently published structure of a trimer comprising Merlin gHgL and VR1814 gO (genotypes gH2 and gO1c) (33) and analysis of gO sequence variability provide a foundation for understanding the potential impact of strain variability on trimer assembly and function.

## RESULTS

### Structure determination and overview of the gH1gLgO1-PDGFRα complex.

**FIG 1:**
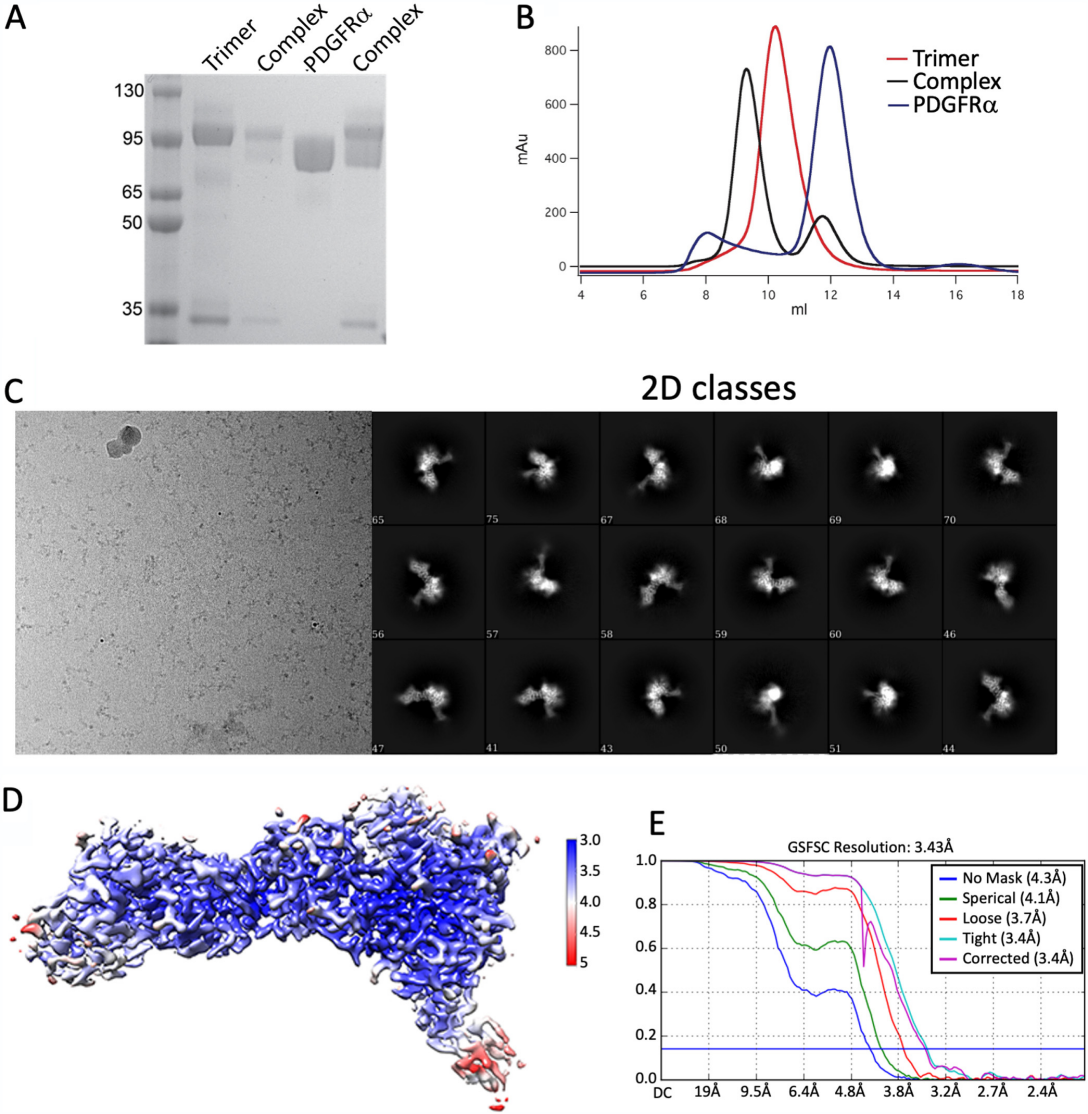
HCMV gHgLgO trimer production, PDGFRα binding, and cryo-EM structure determination. (A) SDS-PAGE of purified trimer, PDGFRα, and the trimer-PDGFRα complex. (B) Isolation of trimer-PDGFRα complexes by gel filtration chromatography. (C) 2D class averages of trimer-PDGFRα complexes. (D) Cryo-EM local resolution map. (E) Gold-standard Fourier shell correlation (GSFSC) curves derived from the final map reconstruction.

We expressed and purified the gHgL proteins derived from the AD169 strain (gH1 genotype) with gO from the TR strain (gO1b genotype) as previously described (Fig. 1A) (34). We similarly expressed the ectodomain of human PDGFRα and made stable trimer-PDGFRα complexes that were isolated by gel filtration chromatography (Fig. 1A and B). Frozen samples were prepared and collected on an FEI Titan Krios microscope. The data were primarily processed using cryoSPARC (35). High-quality two-dimensional (2D) classes were observed, and a 3D reconstruction of the complex was obtained to 3.43 Å resolution (Fig. 1C to E; Fig. S1). Importantly, key regions of the map, including the gO-PDGFRα interface, showed high local resolution in contrast to distal parts of the gH subunit. A model for gO was obtained using rounds of Rosetta *de novo* building and RosettaES (36), followed by manual rebuilding and adjustments. Density of PDGFRα was variable for the extracellular immunoglobulin domains but enabled modeling of the N-terminal three domains and residues at the trimer interface. The final map and model statistics are presented in Table S1.

10.1128/mBio.02625-21.1 FIG S1 Workflow of cryo-EM image processing with cryoSPARC. Download FIG S1, PDF file, 0.3 MB. Copyright © 2021 Liu et al. 2021 Liu et al. https://creativecommons.org/licenses/by/4.0/ This content is distributed under the terms of the Creative Commons Attribution 4.0 International license.

10.1128/mBio.02625-21.9 TABLE S1 Cryo-EM data collection and model refinement. Download Table S1, DOCX file, 0.01 MB. Copyright © 2021 Liu et al. 2021 Liu et al. https://creativecommons.org/licenses/by/4.0/ This content is distributed under the terms of the Creative Commons Attribution 4.0 International license.

**FIG 2:**
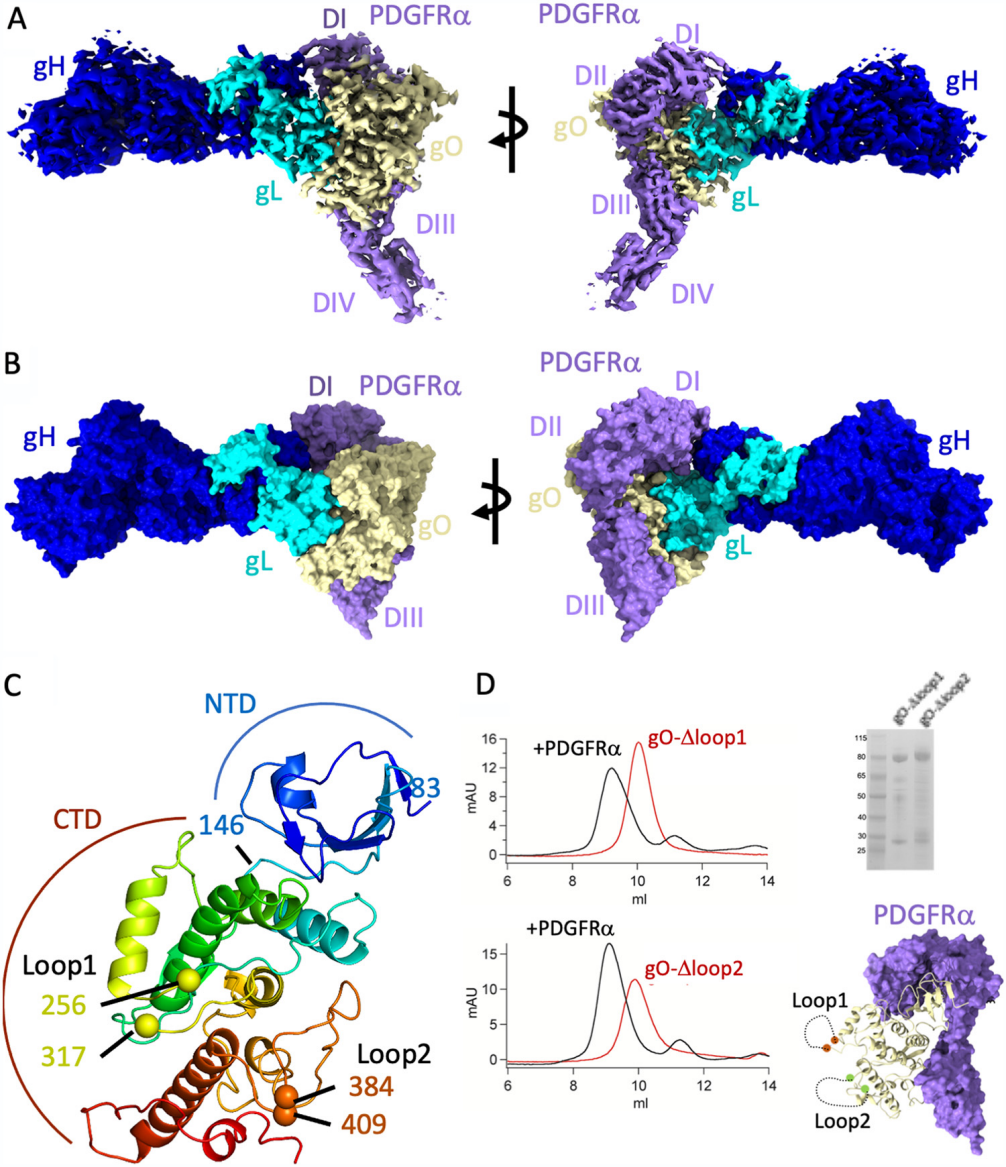
Overview of the trimer-PDGFRα complex. (A) Side views of the EM map of the trimer-PDGFRα complex differing by a 90° rotation, colored by protein. The gH subunit density is blue, gL density is cyan, gO density is pale yellow, and PDGFRα is purple. (B) Side views of the final molecular model of the trimer-PDGFRα complex, colored as in panel A. (C) Cartoon tracing of the gO fold in rainbow coloring from N terminus (blue) to C terminus (red). The NTD and CTD are indicated, as are the positions of the missing loop 1 and loop 2 regions. (D) (Left) Trimer loop 1 and loop 2 mutants bind stably to PDGFRα as observed by gel filtration chromatography. (Right) SDS-PAGE of loop 1 and loop 2 mutants and structural model indicating the positions of the two loops in the gO-PDGFRα complex.

The gHgLgO trimer forms extensive contacts with multiple domains of PDGFRα (Fig. 2A). gO forms a globular lobe attached to one end of the gHgL dimer, and this modular addition to the gHgL core structure provides the majority of the interactions with PDGFRα (Fig. 2A and B). The extracellular region of PDGFRα consists of 5 immunoglobulin-like domains (DI to DV), with domains II and III mediating binding to PDGF ligands. DIV and DV participate in receptor dimerization upon binding natural ligands (32, 37). When bound to the gHgLgO trimer, the three N-terminal domains of PDGFRα (DI to DIII) wrap around gO, with each domain contributing significantly to the interactions with the trimer. DIV is only partially visible in the reconstructions and was not included in the final model (Fig. 2A and B). DIV extends away from the complex toward the presumptive location of the target cell surface. DV was not visible. In total, these gO interactions with PDGFRα bury 2,000 Å^2^ of surface area and involve over 110 amino acids in the gHgLgO trimer and PDGFRα combined. PDGFRα makes additional contacts to gH, but these are more peripheral, burying only 250 Å^2^ and involving only 16 residues. These extensive interactions account for the high binding affinity of the trimer for PDGFRα, which has a *K_d_* of ∼2 nM.

The gO subunit adopts a unique protein fold that is divided into two lobes, an N-terminal beta sheet domain (NTD; residues 83 to 146) (Fig. 2C) and a C-terminal helical domain (CTD; residues 147 to 463 red and orange) (Fig. 2C). Two relatively large loops in gO could not be resolved in our reconstructions, connecting residues 256 to 317 (Fig. 2C, loop 1, yellow spheres) and residues 382 to 409 (Fig. 2C, loop 2, red spheres). The N- and C-terminal residues connecting to these loops are close together in the gO tertiary structure (Fig. 2C), and these amino acid regions are predicted to contain multiple glycosylation sites. The loops are distant from the PDGFRα binding surfaces of gO, suggesting that these loops could be deleted without impacting gO folding and trimer interactions. We generated two loop deletions, expressed the mutant proteins, and observed that they assemble into complexes with PDGFRα similarly to the wild type, consistent with the structural mapping (Fig. 2D). These data indicate that the two glycosylated loop regions are not critical for gO folding, assembly with gHgL, or PDGFRα binding but could play a role in immune evasion through glycosylation.

### gL shows structural plasticity in assembling into pentamer versus trimer complexes.

**FIG 3:**
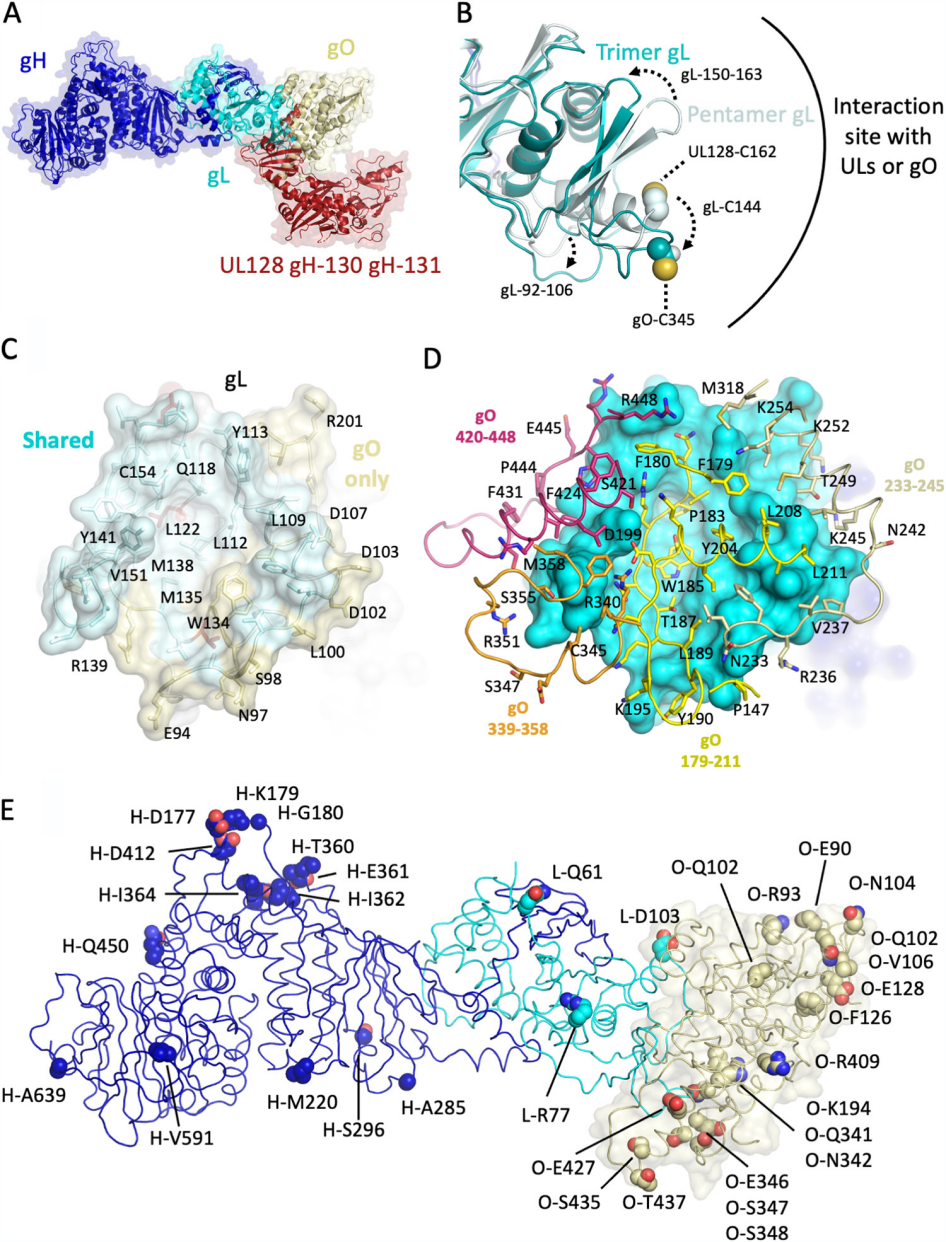
Structural basis for exclusive assembly of trimer or pentamer. (A) Overlay of trimer and pentamer structures. The complexes are shown as cartoons, with surrounding semitransparent surfaces. gH is blue and gL is cyan for both complexes. gO is pale yellow and the UL128–131 subunits in the pentamer are red. (B) Structural changes observed in gL in the trimer and pentamer complexes. Changes in gL residues 150 to 163, the C144 region, and gL 92 to 106 are indicated. The C144 side chain atoms are shown as spheres in the two complexes. (C) Comparison of UL and gO interaction interfaces on gL. The gL surface is shown as semitransparent, with interacting residues shown as sticks. Residues that interact only with gO are in pale yellow, while residues that interact with both gL and UL128–131 are in pale purple. A subset of gL residues that form the central hydrophobic groove and other contacts discussed in the text are labeled. (D) gO uses four CTD segments to assemble onto gL. gL is shown as an opaque surface in cyan, with the four gO segments shown in yellow (residues 179 to 211), pale goldenrod (residues 233 to 254), orange (residues 339 to 358), and pink (residues 420 to 448). (E) Residues that differ in the gH1gLgO1b and gH2gLgO1c trimers are mapped onto the EM structure. Residues are indicated with a prefix for each chain, with H for gH, L for gL and O for gO. Residues are shown as spheres colored by atom type (N, blue; O, red; C, by chain with gH, blue; gL, cyan; gO, pale goldenrod).

HCMV gHgL assembles into pentamers or trimer structures in a mutually exclusive manner, involving a common gL cysteine (C144) that forms covalent bonds with gO C345 or UL128 C162 (38). Superposition of the trimer and pentamer structures shows that the gO (Fig. 3A, yellow) and UL128–131 (Fig. 3A, red) proteins occupy similar positions at the N-terminal end of the gHgL heterodimer. However, the gO protein forms a more extensive interface with gL, burying 2,300 Å^2^ of surface area and involving 118 residues. In comparison, the UL interface with gL buries ∼2,000 Å^2^ and involves fewer contact residues (27).

While the majority of the gHgL structure is similar in the pentamer and trimer complexes, we observed conformational adjustments in gL at the interfaces with the UL and gO proteins that accommodate assembly into the pentamer and trimer (Fig. 3B). Notably, gL residue C144, which makes a disulfide bond with gO and UL 128, is repositioned in the two complexes to form disulfide bonds with UL128 or gO (Fig. 3B). The surrounding gL residues, 137 to 163, also undergo significant changes to accommodate this repositioning of C144. Notably, UL128-C162 is located in a flexible C-terminal extension peripheral to the UL-gL interface. A beta-hairpin structure formed by gL residues 150 to 163 also moves ∼7 to 8 Å to accommodate packing interactions in the trimer and pentamer complexes. We observed additional changes in gL residues 92 to 106, involving an extended chain segment and N-terminal portion of α-helix, which adjust conformation due to gO interactions. These residues are not involved in the UL128–131 interactions in the pentamer.

While the interactions of the ULs and gO with gL are substantially overlapping, each of the complexes involves unique regions (Fig. 3C and Fig. S2). The gL structure forms an extended groove with two gL segments (92 to 110 and 137 to 163) forming ridges on either side of a base formed by residues 118 to 136 (Fig. 3C). A surface representation of gL comparing the interface residues with gO and UL proteins shows many common contacts (Fig. 3C, cyan) and interactions that are unique to gO (Fig. 3C, yellow). UL128–131 also contacts a unique set of residues in gL that are peripheral to the common interaction site (Fig. S2). Both UL128–131 and gO interact with the hydrophobic, central groove on gL, which is lined at the bottom by residues L122 and M138 and on the sides by residues V151, Y141, C154, Q118, and L112. Additional contacts are made by gO with residues W134 and M135 around this central groove. The UL128–131 proteins interact with a peripheral set of residues to one side of the central groove, while gO makes contacts with gL residues on the opposite side (Fig. S2). Interestingly, mutations of gL residues observed here to make unique contacts with gO (R139 and R201) selectively impact trimer and not pentamer function (39).

10.1128/mBio.02625-21.2 FIG S2 Footprints of gO and UL128–131 on gL. Three views comparing UL128–131 and gO footprints on the gHgLgO trimer surface. Interactions that are unique to the UL128–131 complex are shown in dark red, residues that interact with both gL and UL128–131 are shown in pale purple and residues that interact only with gO are colored in pale yellow. Download FIG S2, PDF file, 0.3 MB. Copyright © 2021 Liu et al. 2021 Liu et al. https://creativecommons.org/licenses/by/4.0/ This content is distributed under the terms of the Creative Commons Attribution 4.0 International license.

The interactions with gL are formed by 4 primary gO segments, residues 179 to 211 (Fig. 3D, yellow), 233 to 254 (Fig. 3D, pale yellow), 339 to 358 (Fig. 3D, orange), and 420 to 448 (Fig. 3D, salmon). gO residues 179 to 211 are central to the interface and form a multilayer set of contacts around the gL hydrophobic groove. Residues 179 to 190 form an extended conformation along the length of the gL groove, with gO residues P183, W185, and Y204 occupying the central hydrophobic pocket. Residues 195 to 211 lie above this structure, forming a short beta hairpin and alpha-helical segment that fill in the gL groove. Residues 233 to 254 traverse the outer edge of the gO interface to one side of the groove, forming many of the contacts with gL that are unique to the gO complex compared to UL proteins. The two remaining gO segments (330 to 358 and 420 to 448) interact with gL on the opposite side of the central groove, largely contacting residues that are also involved in UL128–131 interactions.

The gO protein represents one of the more highly variable proteins of the HCMV genome, with strain-specific differences encoding up to 30% differences in gO sequences (20, 21). In contrast, the vast majority of the other ∼200 HCMV proteins exhibit little sequence variability between strains or isolates. These gO sequence variations have also been shown to affect functional differences, impacting cell tropism, virus spreading, and antibody-mediated neutralization (20, 21). Moreover, the trimer and the pentamer are two of the most important targets of neutralizing antibodies (30).

We compared the sequences and structures of the gH1gLgO1b and gH2gLgO1c (33) trimers to gain insight into the distribution of sequence differences that could impact trimer structure and function (Fig. 3E; Fig. S3 and 5). The gHgL subunits align with a root mean square deviation (RMSD) of 0.64 Å over 739 Cα atoms, and the gO aligns with an RMSD of 0.47 over 254 Cα atoms with minor changes in loop regions. Eighteen residue differences could be mapped onto gH, representing differences between the gH1 (AD169) and gH2 (Merlin) genotypes. We also mapped 3 residue changes onto gL and 33 residue changes in gO (Fig. 3E). One of the gL residues that is located at the interface with gO and that differs between AD169 and Merlin is D103 (Fig. 3E). This involves a change from aspartic acid in AD169 to glutamic acid in Merlin. This residue is exclusively involved in gO and not UL interactions and functions along with D102 and D107 to form a negatively charged surface on gL that interacts with gO residues N242, K245, and K252 (Fig. 3C and D). No major differences in gO1b and gO1c amino acids were observed within the gL interface between the two strains, but the amino acids on either side of gO-C345 (residues 341 to 342 and 346 to 348) are not conserved (Fig. 3E), with Merlin (gO5 genotype) and VR1814 (gO1c) sharing identical sequences (Fig. S5). Finally, no gL residue changes between Merlin and AD169 strains are located at the UL interface, although R77 is immediately adjacent to residues that contact ULs (residues 78 to 82). Although gO sequence differences did not cluster at the interface with gL, many changes are dispersed throughout the distal tip of the trimer and in the interface with PDGFRα (Fig. 3E; also, see below).

10.1128/mBio.02625-21.3 FIG S3 Sequence alignment of AD169 and Merlin gH. Residue differences in the Merlin gH are indicated in the lower aligned sequence. Download FIG S3, PDF file, 2.9 MB. Copyright © 2021 Liu et al. 2021 Liu et al. https://creativecommons.org/licenses/by/4.0/ This content is distributed under the terms of the Creative Commons Attribution 4.0 International license.

10.1128/mBio.02625-21.4 FIG S4 Sequence alignment of AD169 and Merlin gL. Residue differences in the Merlin gL are indicated in the lower aligned sequence. Download FIG S4, PDF file, 0.08 MB. Copyright © 2021 Liu et al. 2021 Liu et al. https://creativecommons.org/licenses/by/4.0/ This content is distributed under the terms of the Creative Commons Attribution 4.0 International license.

10.1128/mBio.02625-21.5 FIG S5 Sequence alignment of Merlin, VR1814, and TR gO sequences. The upper colored bar indicates regions of sequence diversity. Conserved residues are shaded in gray. Download FIG S5, PDF file, 0.09 MB. Copyright © 2021 Liu et al. 2021 Liu et al. https://creativecommons.org/licenses/by/4.0/ This content is distributed under the terms of the Creative Commons Attribution 4.0 International license.

We further mapped the sequence variability of 73 gL sequences and 96 gO sequences (Fig. S6) onto the observed gL-gO interface to examine the potential influence of amino acid changes on trimer assembly and function more broadly. Across these sequences, the majority of both gL and gO residues at this interface remain highly conserved, with only moderate variability evident at the periphery of the gO surface involved in interactions with gL (Fig. S6). D103 varies between aspartic acid and glutamic acid in different strains, with ∼50% of each amino acid represented. Some variability in gO maps to the gL C144 loop region of the interface, with corresponding variability in gO-C345-adjacent residues. The highly conserved regions of the gO-gL interface appear important in maintaining the trimer assembly in different strains. It is possible that gH variability could impact gL conformation through indirect conformational dynamic effects.

10.1128/mBio.02625-21.6 FIG S6 Conservation of gL and gO contact surfaces. (A) The gL-gO interface is shown from the perspective of gO. gL is shown as a surface, colored by the level of conservation (red, conserved; blue, variable) in 73 aligned gL sequences. Interacting gO segments are shown in green cartoon format. (B) The gL-gO interface is shown from the perspective of gL. gO is shown as a surface, colored by the level of conservation (red, conserved; blue, variable) in 96 aligned gO sequences. Interacting gL segments are shown in pale green cartoon format. Download FIG S6, PDF file, 0.6 MB. Copyright © 2021 Liu et al. 2021 Liu et al. https://creativecommons.org/licenses/by/4.0/ This content is distributed under the terms of the Creative Commons Attribution 4.0 International license.

### PDGFRα adopts a distinct conformation compared to PDGFRβ bound to PDGF.

**FIG 4:**
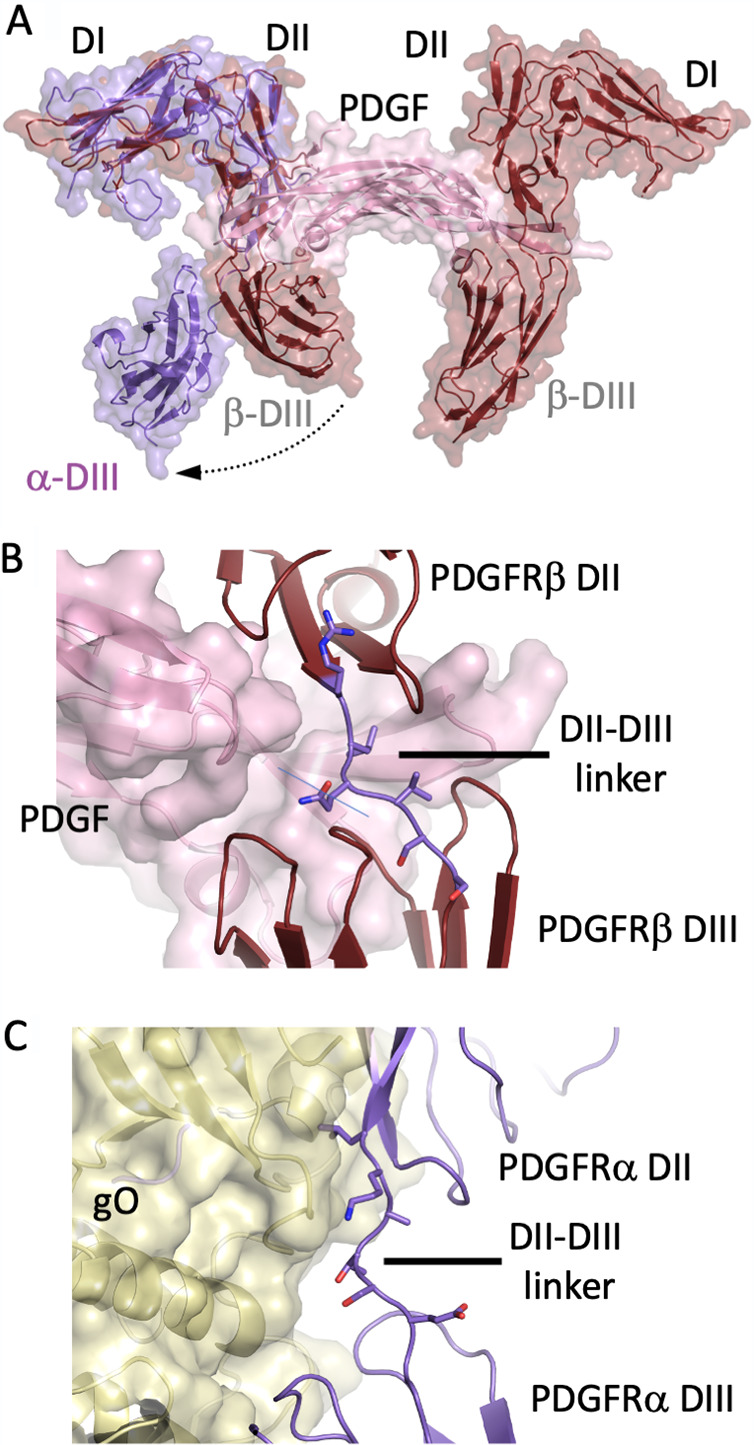
The HCMV trimer induces a PDGFRα conformation that is incompatible with PDGF binding. (A) Overlay of the trimer-PDGFRα complex with the PDGF-PDGFRβ complex through the DI-DII domains of the two receptors. The PDGFRα DIII adopts a distinct conformation when bound to gO compared to PDGFRβ DIII in the complex with PDGF. PDGFRα is in purple, PDGFRβ is in dark red, and the PDGF dimer is in pink. (B) Closeup view of the PDGFRβ DII-DIII linker region and its interactions with PDGF. The two PDGF subunits form a deep groove that interacts across the PDGFRβ DII-DIII linker and restricts the interdomain conformation. (C) Closeup view of the PDGFRα DII-DIII linker region and its interactions with gO. The two distinct PDGFRα DII and DIII domain binding sites on gO result in the separation and reorientation of the two domains.

As noted previously (33), we observed that the PDGFRα conformation when bound to the HCMV trimer is strikingly different from the conformation of the related PDGFRβ bound to PDGF (32) (Fig. 4A). PDGFRβ shares ∼31% identity with PDGFRα and is the closest homolog for which structural information is available. PDGFRα in the conformation bound to the trimer (without the trimer shown) is superimposed on one of the receptors of the entire dimeric PDGFRβ-PDGF complex in Fig. 4A. Overlays of the PDGFRα and PDGFRβ receptor domains demonstrate that the DI-DII conformation is similar in both structures, but there is a substantial difference in the arrangement of the corresponding DIII domains. This conformational difference corresponds to significant displacement of the DIII domain, relative to DI/DII, and an extension of the DII-DIII linker segment leading to a greater separation of the DII/DIII domains. In the PDGFRβ-PDGF complex (Fig. 4B), the dimeric PDGF subunits form a deep pocket that engages both of the DII and DIII domains, as well as the interdomain DII-DIII linker residues, to constrain the conformation of the PDGFRβ-PDGF complex. C-terminal residues in the linker lie along the top surface of PDGFRβ-DIII. In contrast, in the PDGFRα-gO complex (Fig. 4C), the gO contact surfaces with the two PDGFRα domains are spatially separated and the interdomain DII-DIII linker traverses more peripherally along the gO surface in an extended conformation (Fig. 4C). Nonetheless, the PDGFRα and PDGFRβ receptors use substantially overlapping residues in their domains to bind gO or PDGF, despite these observed conformational differences.

### gO binds to PDGFRα through three distinct contact interfaces.

**FIG 5:**
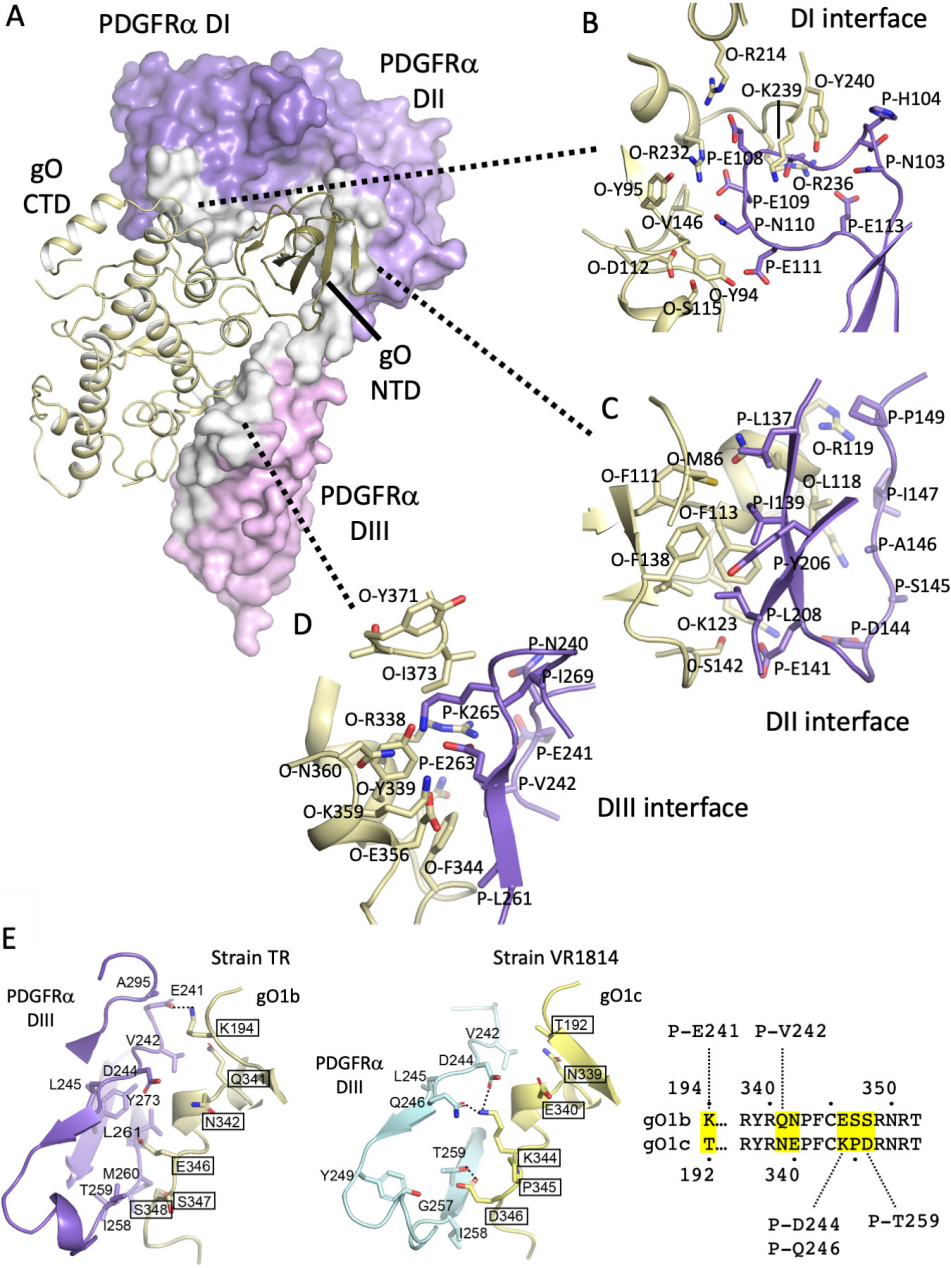
Structural analysis of the gO-PDGFRα binding interface. (A) PDGFRα DI, DII, and DIII form extensive interactions with the trimer through three contact sites on gO. PDGFRα is represented as a surface colored in shades of purple for each domain, with DI the darkest, DII intermediate, and DIII the lightest. Residues in PDGFRα in the interfaces with gO are highlighted in white. gO is shown in cartoon format, with the NTD in sand and the CTD in pale yellow. The three distinct interfaces between gO and PDGFRα are indicated by the dotted lines connecting to panels B to D. (B) Detailed view of interactions at the gO interface with DI. DI residues derived from PDGFRα loop, consisting of residues 103 to 113, while gO residues derived from a combination of NTD and CTD regions. gO residues are colored by atom, with carbon atoms in pale yellow. PDGFRα residues are colored by atom, with carbon atoms in purple. (C) Detailed view of interactions at the gO interface with DII. (D) Detailed view of interactions at the gO interface with DIII. (E) Comparison of sequence differences in gO1c (left) and the gO1b (right) interfaces with DIII. A sequence alignment of the gO strains is shown to the right with variable amino acids highlighted in yellow with their contacts to PDGFRα indicated above and below the alignment.

Three different surfaces of gO1b interact with each of the three N-terminal PDGFRα domains (DI-DIII), involving 61 gO and 55 PDGFRα residues. PDGFRα domains wrap tightly and extensively like a human hand around the gO, contacting two CTD sites on either side of a central NTD-DII interaction (Fig. 5A). PDGFRα DI also contacts residues in gH, although these interactions are much less extensive (Fig. S7). The contacts between gH and PDGFRα are centered on a salt bridge between gH-R48 and PDGFRα-E52 and involve the N termini of both proteins. The relatively limited contacts between gH and PDGFRα are consistent with the essential role of gO in determining PDGFRα binding.

10.1128/mBio.02625-21.7 FIG S7 PDGFRα interactions with gH. (A) gH interactions with PDGFRα DI. PDGFR α is represented as a surface colored in shades of purple for each domain, with DI the darkest, DII intermediate and DIII the lightest. Residues in PDGFRα in the interface with gH are highlighted in white. gHgLgO is shown in cartoon format and colored blue for gH, cyan for gL, and pale yellow for gO. The dotted line indicates the region of contact shown in panel B. (B) Detailed view of interactions at the gH interface with DI. gH residues are colored by atom, with carbon atoms in blue. PDGFRα residues are colored by atom, with carbon atoms in purple. Download FIG S7, PDF file, 3.0 MB. Copyright © 2021 Liu et al. 2021 Liu et al. https://creativecommons.org/licenses/by/4.0/ This content is distributed under the terms of the Creative Commons Attribution 4.0 International license.

A concave surface at the junctions of the gO NTD and CTD domains contacts a protruding loop in the PDGFRα DI (Fig. 5A and B). This interaction is dominated by a negatively charged loop in PDGFRα DI (103 to 113), containing residues E108, E109, N110, E111, and E113 (Fig. 5B). Positively charged residues in gO that complement this PDGFRα loop are gO-R214, gO-R232, gO-R236, and gO-K239. The charge interactions between gO-R232 with PDGFRα-E109 and gO-R214 with PDGFRα-E108 are within 2.7 to 3.3 Å and likely stabilize this interface.

The PDGFRα DII is contacted by the tip of the gO NTD, which packs against a face of the PDGFRα DII Ig-like domain and the DII-DIII linker. This interface is dominated by a cluster of hydrophobic interactions in gO (M86, F111, F113, and F138) (Fig. 5C) that contact PDGFRα residues L137, I139, Y206, and L208. Polar contacts, including O-K123, O-S142, P-D144, and P-E141, flank the hydrophobic pocket. DIII is contacted by the gO CTD (amino acids [aa] 338 to 373), which interacts with the edge of a beta-sheet in DIII, with residues in the Ig domain C and D strands and BC and DE loops making up the majority of the binding interactions (Fig. 5D). This site shows a mixed set of interactions involving both hydrophobic and buried charges (Fig. 5D). The charge interactions involve a centrally located interaction between O-R338 with P-E263. This salt bridge is stabilized by O-Y339, which hydrogen bonds to P-E263. A cluster of charged residues (O-E356, O-K359, and P-K265) flanks these central contacts. Also involved are hydrophobic contacts: O-F344, O-P242, and P-L261. These three separate surfaces of gO contacting three surfaces of PDGFRα explain the high affinity of trimer (2 × 10^−9^) for PDGFRα.

We compared the sequences and structures of the gO1b-PDGFRα and gO1c PDGFRα complexes and found that unlike the gL-gO interface, where the gO sequences are highly conserved, the gO sequences show variation at the gO-PDGFRα DIII interface (Fig. 5E). Six residues differ between the two strains, with most of these mapping to a contiguous segment of residues 341 to 348 (TR numbering). gO1b has a positively charged Lys at 194 which forms salt bridge with the negatively charged E241 in PDGFRα, but gO1c has a Thr (T192) at this position and does not make this salt bridge (Fig. 5E). However, gO1c makes three additional salt bridge and hydrogen bond interactions with PDGFRα through substitutions at residues K344 (E346 in gO1b) and D346 (S348 in gO1b) that are absent in gO1b (Fig. 5E). Other variable residues make limited or no contacts across the interface, except gO1b N339 with PDGFRα V242. Overall, these multiple charge changes at the gO-PDGFRα interface could affect receptor binding affinity, but the sequence differences are readily accommodated by small adjustments in interface residues. This gO variability in the interface with PDGFRα DIII contrasts with the higher conservation of gO amino acids in contact with DI and DII.

Since gO polymorphisms have been shown to influence the efficiency and selectivity of both cell-free infection and cell-cell spreading of HCMV (21, 40, 41), we examined gO conservation in 96 strain sequences at the PDGFRα interfaces. In contrast to the conservation observed at the gO-gL interface, gO sequence variation is significantly greater at the interface with PDGFRα (Fig. S8) as well as in adjacent gO surfaces. We observed variability in the PDGFRα DI and DIII contacts (Fig. S8), overlapping with the differences observed comparing gO1b-PDGFRα with gO1c-PDGFRα. This gO variability may impact binding to PDGFRα, virion attachment, and gB fusion activation.

10.1128/mBio.02625-21.8 FIG S8 Conservation of gO at PDGFRα interfaces. (A) The gO interfaces with PDGFRα DI and DII are shown. (B) The gO interfaces with PDGFRα DII and DIII are shown. gO is shown as a surface, colored by the level of conservation (red, conserved; blue, variable) in 96 aligned gO sequences. Interacting PDGFRα domains are shown in cartoon format, colored purple. Download FIG S8, PDF file, 0.7 MB. Copyright © 2021 Liu et al. 2021 Liu et al. https://creativecommons.org/licenses/by/4.0/ This content is distributed under the terms of the Creative Commons Attribution 4.0 International license.

### Mutational analysis of the gO interface with PDGFRα.

**FIG 6:**
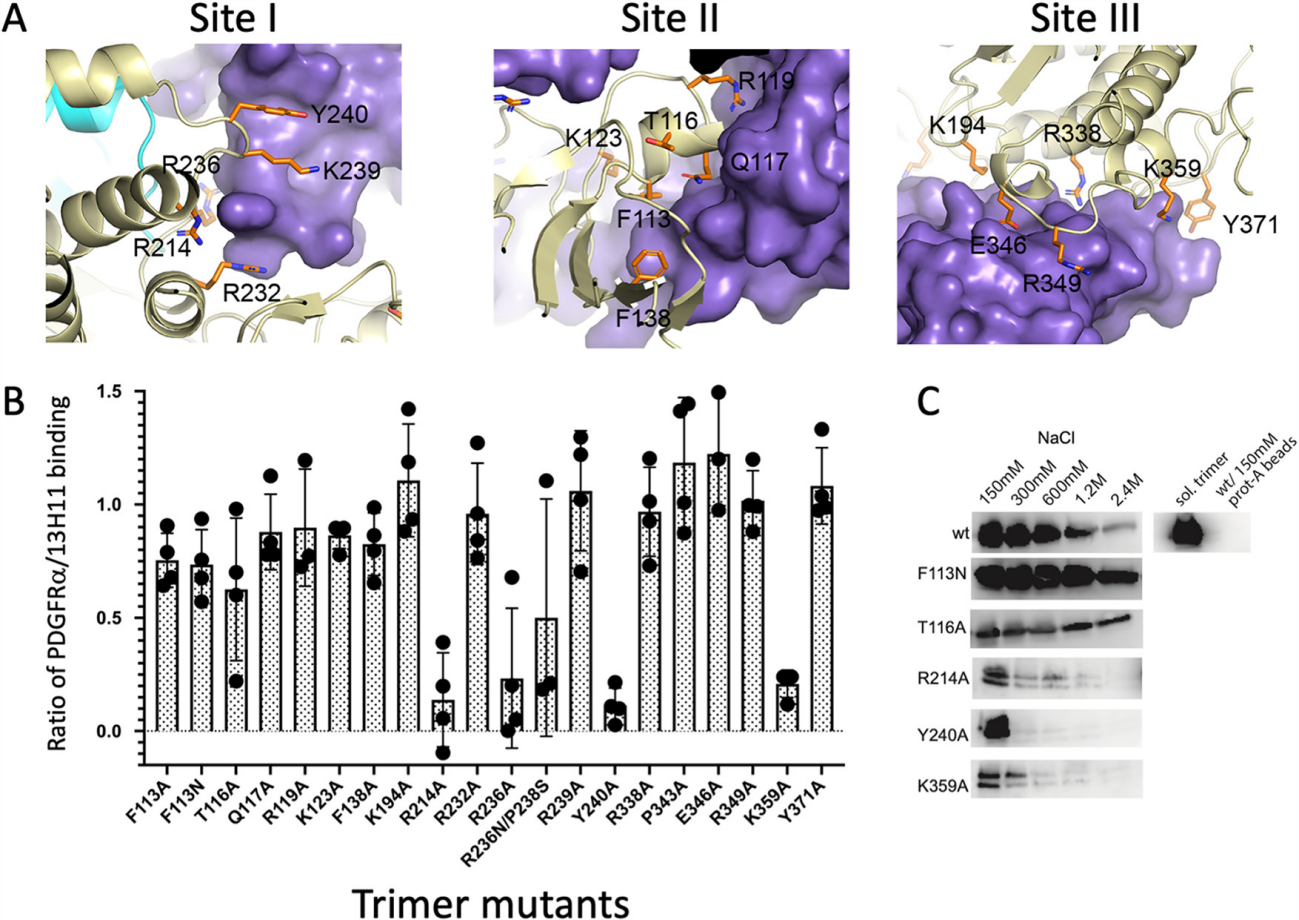
Mutagenesis of gO interfaces with PDGFRα. (A) Locations of gO residues in the three interfaces with PDGFRα selected for mutational analysis. (B) Cell-based binding studies of gO trimer mutants, showing the ratio of 13H11 and PDGFRα binding. (C) Coimmunoprecipitation studies of selected gO trimer mutants with PDGFRα.

To characterize gO residues that contact PDGFRα, we generated a panel of 20 gO substitution mutations shown in Fig. 6. We examined whether these had an impact on trimer expression and PDGFRα binding. 293E cells were transfected with gO wild type (wt) or gO mutants along with gL and full-length gH. Cells were stained with an scFv form of the gH 13H11 antibody and in parallel with a soluble form of PDGFRα (Fig. 6B). 13H11 binds to the C-terminal region of gH in both trimer and pentamer complexes and was used to monitor gH surface expression. The ratios of 13H11 to PDGFRα staining were calculated as a measure of mutant trimer binding to PDGFRα.

Most of the mutations had limited impact on the binding of PDGFRα to gH1gLgO1b, indicating that the 3 distinct interaction surfaces resist disruption of high-affinity receptor binding when only single amino acid changes are made, as was observed with gH2gLgO1c (33). Mutants that exhibited more than 66% of wild-type (wt) binding were considered to have mild or no impact on PDGFRα binding. The R236N/P238S mutant fell into an intermediate category, reducing binding to ∼50% of wt. Since this mutation was designed to introduce a glycosylation site at R236, partial glycosylation could account for the partial block in PDGFRα binding. Four mutations reduced binding of PDGFRα to less than ∼33% of wt (R214A, R236A, Y240A, and K359A). R214, R236, and Y240 are located in the interface with PDGFRα DI. R214 and R236 contribute to the charge complementarity of the negatively charged PDGFRα DI loop (Fig. 5B and 6A), with R214 forming a salt bridge with PDGFRα-E108. Y240 caps off one edge of the DI interaction surface (Fig. 5B and 6A). K359 is located in the DII interface, is involved in a network of charge-charge interactions across the interface, and forms a salt bridge with gO-E356 (Fig. 5D and 6A).

Given the extensive binding interactions of gO with PDGFRα, we tested a subset of these mutations (F113N, T116A, R214A, Y240, and K359A) in pulldown assays, in the presence of increasing salt concentrations to probe trimer-PDGFRα complex stability. 293E cells were cotransfected with wild-type gO (34) or mutant forms of gO and soluble gH and gL. Cell culture supernatants were collected and incubated with cell culture supernatants containing soluble PDGFRα with an IgG Fc tag (42). The PDGFRα-Fc complex was pulled down with protein A-agarose and in the presence of increasing salt concentrations from 150 mM to 2.4 M. The wild-type gO trimer formed complexes with PDGFRα that are relatively stable at 300 mM NaCl, but there was diminished binding at 1.2 and 2.4 M NaCl (Fig. 6C). The F113N mutant forms complexes that are stable to at least 1.2 M NaCl, unlike the wt, while the cell surface binding assays indicated binding similar to that of the wt. Expression of the T116A gO was lower, but this trimer complex displayed stability at high concentrations of salt. T116 is located in the DII interface. Three gO mutants implicated by cell binding experiments as having weaker interactions with PDGFRα (R214A, Y240A, and K359A) all showed increased sensitivity to higher salt concentrations in the pulldown assays (Fig. 6C). Notably, these three mutations would affect charge-charge interactions at the interface, consistent with their increased sensitivity to NaCl. The R214A and K359A mutations directly remove charge-charge interactions at the interface, which would destabilize binding and reduce long-range interactions that may be affected by high salt. The Y240A mutation may act indirectly by destabilizing the gO interface with PDGFRα DI, which involves many charge-charge interactions. These experiments provided further confirmation of the impact of R214A, Y240A, and K359A gO mutants on PDGFRα binding.

## DISCUSSION

We determined the structure of the HCMV gH1gLgO1b trimer bound to PDGFRα using cryo-EM and generated gO mutants to interrogate the interactions between gO and receptor. These studies enabled comparisons with another recently determined structure of the HCMV gH2gLgO1c trimer bound to PDGFRα. Overall, these studies highlight key features of the gHgLgO trimer that further inform our understanding of its assembly and function in HCMV entry.

The trimer gO subunit adopts a unique protein fold with a two-domain structure consisting of a smaller beta-sheet-rich NTD and a primarily helical CTD. Assembly of gO onto gHgL occurs through extensive interactions with the gL subunit that involve covalent tethering through a disulfide bond between gO-C345 and gL-C144. The assembly of gHgL onto gO occurs through an interface on gL that largely overlaps the face of gL that interacts with UL128–131. However, gL shows conformational plasticity in its assembly into these distinct complexes, adjusting its structure to accommodate gO or UL128–131 proteins. In addition, gO forms more extensive and more unique interactions with gL than UL128–131 subunits. gO engages a large hydrophobic groove in gL and uses 4 contiguous segments within its CTD to encapsulate one end of the gHgL heterodimer. These gO-gL interactions are highly conserved across many HCMV strains, indicating that sequence differences at the gO-gL interface in both proteins may not play a major role in determining the efficiency of strain-dependent trimer assembly, consistent with functional experiments swapping gO alleles (21, 40, 43, 44). We observed in our mutant studies that gO sequence changes distant from the gO-gL interface significantly decreased expression levels of cell surface and secreted trimer. It may be that certain strains could encode gO variants that behave similarly to our gO mutants, with amino acid changes distant from the gL interface impacting trimer expression levels.

We also demonstrated that two gO loops, which are apparently highly glycosylated, can be deleted without disrupting PDGFRα interactions. The structural studies map these loops to a surface of gO that is oriented away from the receptor interaction interface, and these loop regions were not visible in either of the two trimer structures. These loops may play a role in immune evasion, similar to the glycosylated loops in HIV Env, protecting the HCMV trimer from antibody recognition, as has been suggested recently (21). We have demonstrated that the loops are dispensable for binding to PDGFRα, but it remains to be tested whether the deletions impact other trimer functions during viral entry. It will be interesting to establish whether these loops act to protect the trimer from neutralizing antibodies, similar to the variable loops in HIV gp120.

PDGF dimers bind at the junction of the receptor DII-DIII domains, interacting with the C-terminal end of DII and N-terminal end of DIII and restricting the receptor to an L-shaped conformation (32). In contrast, the trimer interactions with PDGFRα cause a major structural bending of the DII and DIII domains, so that PDGFRα forms an inverted C structure, wrapping around gO. This conformational change requires a repositioning of the DII and DIII domains relative to each other. The interactions of PDGFRα DI to DIII are thereby spread over three contact sites with gO, along with a relatively minor contact of DI with gH that involves far fewer residues. Interestingly, the gO interfaces with PDGFRα DII and DIII involve receptor residues that are likely also involved in PDGF dimer binding, despite gO binding requiring a distinct receptor conformation. The gO interactions with PDGFRα DI are unique to the HCMV trimer complex and are made by residues at the junction of the gO NTD and CTD. The gO NTD plays a central role in PDGFRα interactions, forming the primary contacts within the DII interface and also contributing to the DI interface, consistent with prior mapping experiments that implicated the gO N-terminal region (45). This contrasts with the dominant role of the gO CTD in interactions with gL. Interestingly, gO sequence variability includes residues involved in PDGFRα interactions, in both the DI and DIII contact interfaces, suggesting that trimers derived from certain HCMV strains may exhibit alterations in receptor-binding affinity.

The trimer binds to PDGFRα with nanomolar affinity, which is explained by the extensive interactions formed over three distinct surfaces of gO that interact with PDGFRα. HCMV may benefit from remaining tightly bound to PDGFRα because entry into fibroblasts involves trafficking in the plane of the plasma membrane to sites where macropinocytosis occurs, followed by fusion within endosomes (46, 47). The three extracellular PDGFRα Ig-like domains are required for HCMV entry into fibroblasts, but the intracellular kinase domain is dispensable, indicating that PDGFRα signaling is not essential for internalization (17). To characterize the relative contribution of the three gO surfaces that contact PDGFRα, we constructed a panel of gO substitution mutations. The majority of the single point mutants generated had minimal impact on receptor binding. Similarly, Kschonsak and colleagues produced point mutations in each of the gO DI, DII, and DIII binding domains that added a bulky N-linked oligosaccharide, and none of these mutations significantly reduced PDGFRα binding (33). They concluded that it was necessary to combine mutations in more than one gO contact domain in order to substantially affect PDGFRα binding. However, we characterized a larger number of alanine substitution mutations and identified four mutations of residues located in the DI and DII interfaces that substantially reduced the stability of the PDGFRα interaction. These data highlight the resiliency of the trimer-PDGFRα complex to disruption but also indicate that the gO contacts with DI and DII may be more critical to receptor binding. Amino acid substitutions present in different gO strains may well impact receptor binding affinity and HCMV entry and may also impact the neutralizing breadth of anti-trimer antibodies.

How HCMV pentamers and trimers activate the gB protein for entry into cells remains to be determined. The lack of receptor-induced conformational changes in the gHgL subunits of the trimer relative to previously determined pentamer structures indicates that other mechanisms are needed to explain gB activation. For example, gB activation might depend on clustering of trimer and/or pentamer receptor complexes, as observed for the two-component Nipah virus entry mechanism (48). gHgL-gB complexes have been observed in coimmunoprecipitation experiments (26), and in cryo-EM tomograms of HCMV virions (25), a potential complex of gHgL with prefusion gB was identified. Models for this gB-gHgL complex, based on the recently determined prefusion gB structure (11), indicate potential interactions between the gH C-terminal domain and gB. In this model, reorientation of the gHgLgO trimer relative to the membrane or lateral clustering of trimers induced by receptor binding could provide a signal to activate gB-mediate membrane fusion and virus entry.

## MATERIALS AND METHODS

### Protein expression and purification.

The construction, expression, and purification of the HCMV trimer were described previously (34) and were carried out with minor modifications. The gH and gL constructs were subcloned from the original pYD7 vector into the pTT5 vector to ensure the optimal expression of the trimer in 293-6E cells (49). The gH, gL, and gO vectors were cotransfected into HEK6E cells, and the supernatant was harvested at day 7 for purification. The supernatant was first passed through Ni-affinity resin pre-equilibrated with 300 mM NaCl, 20 mM Tris-HCl (pH 8.0), and 5 mM imidazole, and the protein was eluted from the beads with 300 mM NaCl, 20 mM Tris-HCl (pH 8.0); 300 mM imidazole. EDTA (10 mM) was added, and the eluates were concentrated to ∼1 mg/ml. The concentrated protein was loaded onto Strep-Tactin XT resin pre-equilibrated with 300 mM NaCl and 20 mM HEPES (pH 7.5) (buffer S). The protein was eluted with 300 mM NaCl, 20 mM HEPES (pH 7.5), and 100 mM biotin after extensive washing with buffer S. The protein was concentrated to ∼1 mg/ml and loaded to a Superdex 200 10/300 GL column pre-equilibrated with buffer S, and the peak at ∼10 ml was collected. The construction, expression, and purification of PDGFRα were also described previously (42). The PDGFRα ectodomain was expressed with a tobacco etch virus (TEV)-cleavable Fc tag in HEK6E cells. The supernatant was harvested, applied to protein A resin pre-equilibrated with buffer S, and washed extensively with buffer S. The protein-bound resin was then incubated overnight with TEV protease to release PDGFRα. The elution was concentrated to ∼2 to 3 mg/ml and loaded onto a Superdex 200 10/300 GL column pre-equilibrated with buffer S, and the peak at ∼11.7 ml was collected. To make the trimer-PDGFRα complex, purified trimer and PDGFRα were combined in a 1:2 ratio, incubated on ice for 1 h, and then injected onto a Superdex 200 10/300 GL column. The central fractions of the complex peak were collected and concentrated to ∼1.6 mg/ml.

### Cryo-EM data collection.

For cryo-EM data collection, 3 μl of trimer-PDGFRα complex at ∼1.6 mg/ml with 0.05% octyl-β-d-glucopyranoside was applied to glow-discharged Quantifoil gold R2/1 200 mesh grids. The grids were blotted with Whatman filter paper for 2 s at 96% humidity using a Leica GP automatic plunge freezer and frozen in liquid ethane. A total of 13,413 movie stacks were collected on an FEI Titan Krios electron microscope operated at 300 kV with an energy filter (20-keV slit width) and a Gatan K2 Summit direct detector over two data collection sessions. Movie stacks were recorded at ×130,000 magnification, corresponding to 1.06 Å/pixel, with a total accumulated dose of 75 e Å^−2^, 0.2 s/frame, and a total exposure time of 10 s.

The cryo-EM data were processed primarily in cryoSPARC (35). Each data set was processed individually to a high-resolution structure, and then the two particle sets were combined for a final round of image processing and reconstruction. The image stacks were motion corrected by patch motion, and the contrast transfer function (CTF) was estimated using patch CTF estimation in cryoSPARC. A previously collected lower-resolution data set was used to generate 2D templates for automatic picking in Relion (50), after manual picking of ∼2,000 particles. We imported these 2D templates into cryoSPARC to allow template picking on the two higher-resolution data sets. The picked particles were inspected, extracted with a box size of 360 pixels, and then Fourier cropped to a box size of 120 pixels. After a few rounds of 2D classification to remove junk particles, the remaining good particles were extracted again with a box size of 360 pixels and no cropping. A few rounds of *ab initio* heterogenous refinement and homogeneous refinement were used to further clean and classify the particles. Once the map quality and resolution could no longer be improved by further reducing the particle numbers, we performed local and global CTF refinement on the subset of the best particles and reran the homogenous and nonuniform refinement steps. We combined the best subset of particles from the two data sets and performed an additional round of *ab initio* heterogenous and homogeneous refinement. Local refinement was performed using a mask covering either gHgL or gO-PDGFRα regions to further improve the map.

### Trimer-PDGFRα model building and refinement.

The previously determined pentamer gHgL^Merlin^ (gH2gL) structure (5VOB) (27) was used to generate the trimer gHgL^AD169^ (gH1gL) model using Rosetta comparative modeling (Rosetta CM) tool (51). The PDGFRα model was generated using the I-TASSER server (52). The two models were docked into the EM density map using UCSF Chimera (53). DI-DII and DIII of PDGFRα were docked into the density map separately, saved as one model, and then reconnected in Coot (54). gO density was segmented from the entire density map in Chimera using segger with the gHgL and PDGFRα models. A partial gO model was generated after several rounds of Rosetta *de novo* model building guided by the gO density map with some manual editing (36). RosettaES and Rosetta CM were used to complete the gO model (36). A combination of Rosetta refinement, Phenix refinement (55), and UCSF ChimeraX-ISOLDE (56) and Coot were used to further refine the model. N-linked glycans were built onto the model using the Coot carbohydrate module.

### Construction of gO mutants.

BioLuminate (Schrodinger) (57) alanine scanning was performed on the gO residues at the interfaces with PDGFRα. For each of the three binding interfaces, we chose to mutate amino acids to alanine that had the largest predicted change in binding affinity. Point mutations were introduced into the gO open reading frame (ORF) (strain TR) using the Q5 site-directed mutagenesis kit (New England Biolabs) with wt gO cloned into the pTT5 expression plasmid as the template (34). The primers used were F113N-fwd-GACAACTACAGCACCCAGC, F113N-rev-GAACCACAGGTAGGTCACG, T116A-fwd-CAGCGCCCAGCTGCGGAAG, T116A-rev-TAGAAGTCGAACCACAGG, R214A-fwd-CTGGCTTACGCCCAGCGG, R214A-rev-CAGCAGGGCGGTCAGGC, Y240A-fwd-AGGCAATCAACGGCACCAA, Y240A-rev-TGGGCACCCGGAACAGG, K359A-fwd-TGGCGAATACCCACGTGC, and K359A-rev-TGAACTCGCTCACGGCGG. Clones were isolated and the individual mutations were sequence verified.

### Cell-based binding studies of gO mutants.

The full-length gH (flgH) construct was generated from the secreted gH construct by appending the gene sequence of the transmembrane and intracellular domain via insertion mutagenesis. The primers (forward, ggcatctacctgctgtaccggatgctgaaaacctgcgcaTCACTGGTACCAAGGGGC, and reverse, gatgatggcagacagggcgtacacgtcatcatcagcagACGTGAATCTGTAGCATCAACG) were designed in NEBbase changer. In these primers, the lowercase letters are the inserted sequence and the capitalized letters represent the sequences within the original secreted gH construct. flgH, gL, and gO wild-type and mutant plasmids were cotransfected into HEK293-6E cells at a 1:1:1 ratio. The cells were harvested ∼64 h posttransfection by centrifuging at 400 × *g* for 5 min and washed twice with an equal volume of phosphate-buffered saline (PBS) buffer. Cells were resuspended in PBS, and 20 μl of the cells was pipetted into V-bottom 96-well plates. Just before addition of the staining reagents, the cells were centrifuged at 400 × *g* for 5 min to remove the PBS buffer. Untransfected HEK293-6E cells were used as the negative control. PDGFRα and 13H11 (29) were biotinylated using EZ-link *N*-hydroxysuccinimidobiotin (Thermo Fisher) following the manufacturer’s protocol. One hundred microliters of 4 μg/ml biotinylated PDGFRα or 13H11 in PBS buffer with 0.5% bovine serum albumin (BSA) was added to cells and incubated in a microplate shaker at room temperature for half an hour at 900 rpm. Fifty microliters of 1.6% paraformaldehyde (PFA), freshly diluted from 16% using PBS, was added to the cells, and the plates were covered with foil and incubated on ice for 10 min. The cells were centrifuged at 400 × *g* for 5 min to remove the supernatant and washed twice with 160 μl of PBS. The cells were resuspended in 100 μl of a 1:400 dilution of Alexa Fluor 647-streptavidin in PBS with 0.5% BSA and incubated in a microplate shaker at room temperature for half an hour at 900 rpm. Fifty microliters of 1.6% PFA was added to each well and incubated on ice as described above. The cells were centrifuged to remove the supernatant, washed twice with PBS buffer, and resuspended in 100 μl of PBS buffer. The cells were analyzed using an Accuri C6 flow cytometer, and ∼2,000 live cell events were collected from each well.

### Coimmunoprecipitation of gO mutants.

gO mutant plasmids along with wild-type soluble gH and gL (34) were transfected into 25-ml cultures of 293-6E cells using Lipofectamine (Thermo Fisher) according to the manufacturer’s instructions. At 3 days posttransfection, the cell culture supernatants were harvested, clarified by centrifugation at 1,000 × *g* for 5 min, and then concentrated to 5 ml using Amicon Ultra filtration devices. For immunoprecipitations, 1 ml of supernatant was supplemented with different concentrations of NaCl (150 mM to 2.4 M) and then incubated with 50 μl of protein-A agarose that was coupled to a soluble version of PDGFRα fused to an FC domain (42) for 1 h at 4°C while rotating. The protein-A agarose was then washed with 20 mM Tris-HCl (pH 7.4)–NaCl (150 mM to 2.4 M) buffer 5 times for 3 min each at 4°C while rotating. The beads were then suspended in SDS-PAGE gel loading buffer (50 mM Tris [pH 6.8], 10% glycerol, and 2% SDS) with 1% 2-mercaptoethanol. The precipitated proteins were separated using SDS-polyacrylamide electrophoresis and then transferred to polyvinylidene fluoride (PVDF) membranes. Membranes were incubated in Tris-buffered saline containing 0.1% Tween 20 (TBST) plus 5% nonfat milk, washed, and incubated in TBST and a rabbit polyclonal serum specific for gL (1:1,000) (58) for 1 h at 4°C. Membranes were washed 3 times for 10 min in TBST and incubated in TBST with horseradish peroxidase-conjugated secondary antibodies for 1 h. Proteins were detected by incubating membranes in chemiluminescent reagent (Perkin Elmer) and imaged with an ImageQuant LAS 4000 system (GE Healthcare).

10.1128/mBio.02625-21.10 TEXT S1 UniProt accession numbers for gH, gL, and gO sequences used for sequence variability analysis. Download Text S1, DOCX file, 0.01 MB. Copyright © 2021 Liu et al. 2021 Liu et al. https://creativecommons.org/licenses/by/4.0/ This content is distributed under the terms of the Creative Commons Attribution 4.0 International license.
