# Supplementary figures and images for: Cryo-Electron Microscopy Structure and Interactions of the Human Cytomegalovirus gHgLgO Trimer with Platelet-Derived Growth Factor Receptor Alpha
Source: mBio. 2021 Oct 26;12(5):e02625-21. doi: 10.1128/mBio.02625-21 (PMC8546573; doi:10.1128/mBio.02625-21)

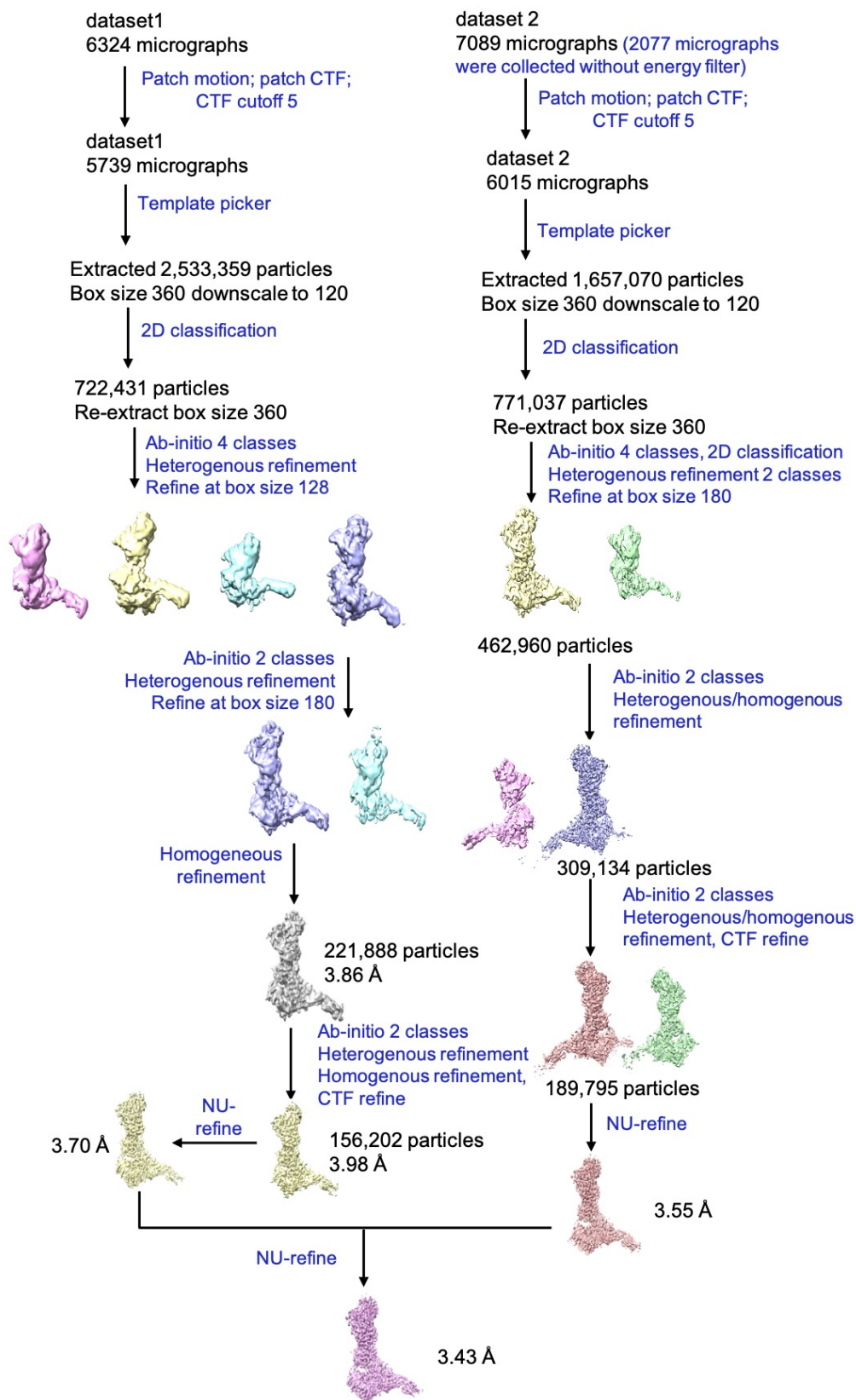

Supplementary Figure 1. Workflow of Cryo-EM image processing

Supplement: FIG S1 [file mbio.02625-21-sf001.pdf]

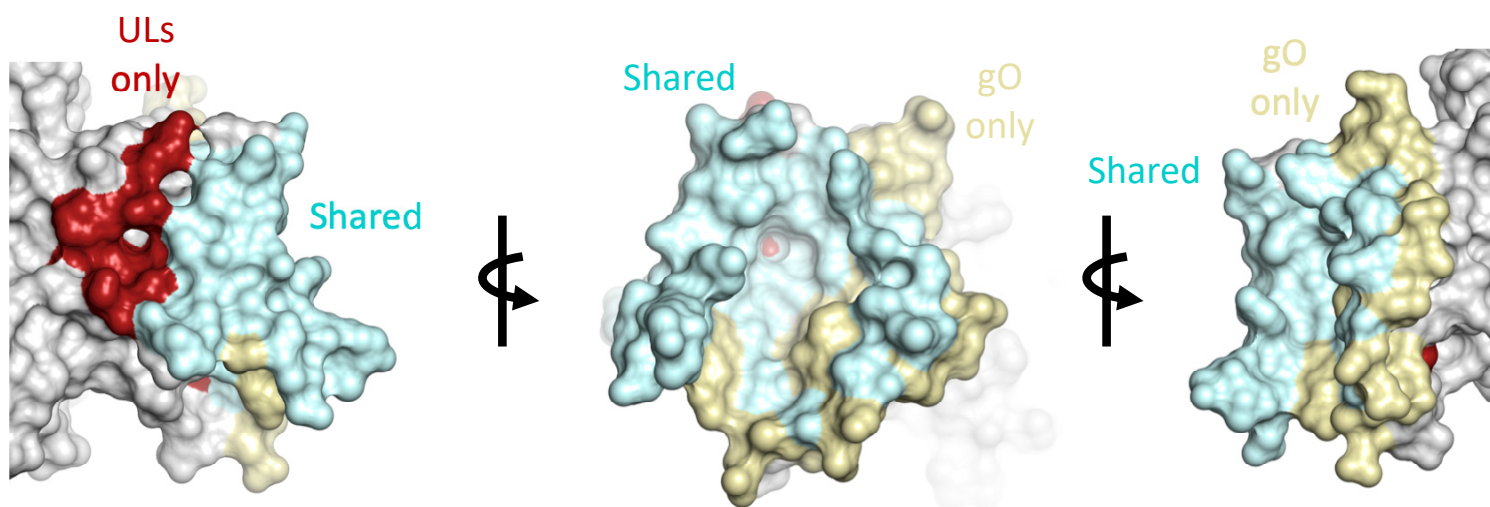

Supplementary Figure 2. Footprints of gO and UL128-131 on gL

Supplement: FIG S2 [file mbio.02625-21-sf002.pdf]

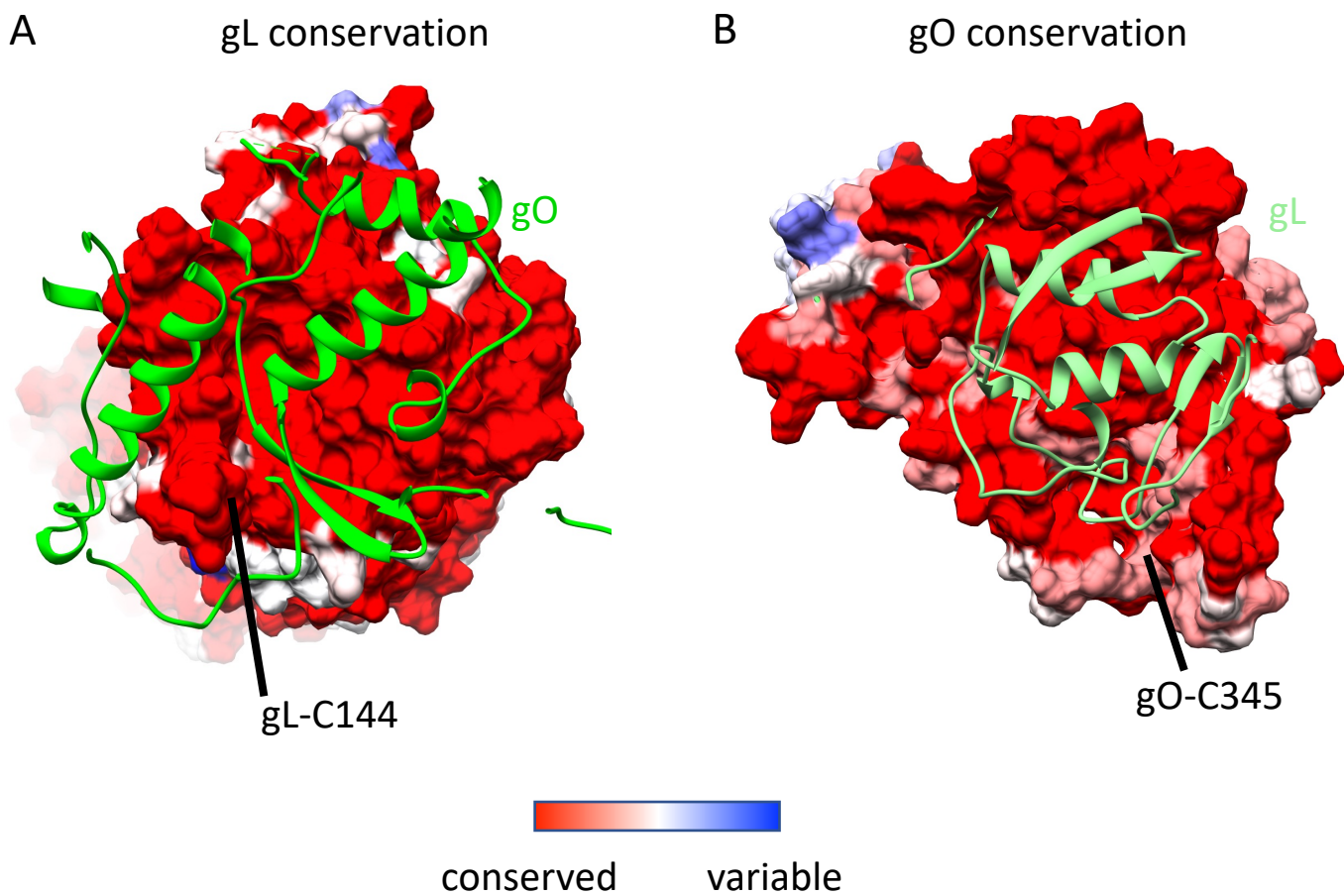

Supplementary Figure 6. Conservation of gL and gO contact surfaces

Supplement: FIG S6 [file mbio.02625-21-sf006.pdf]

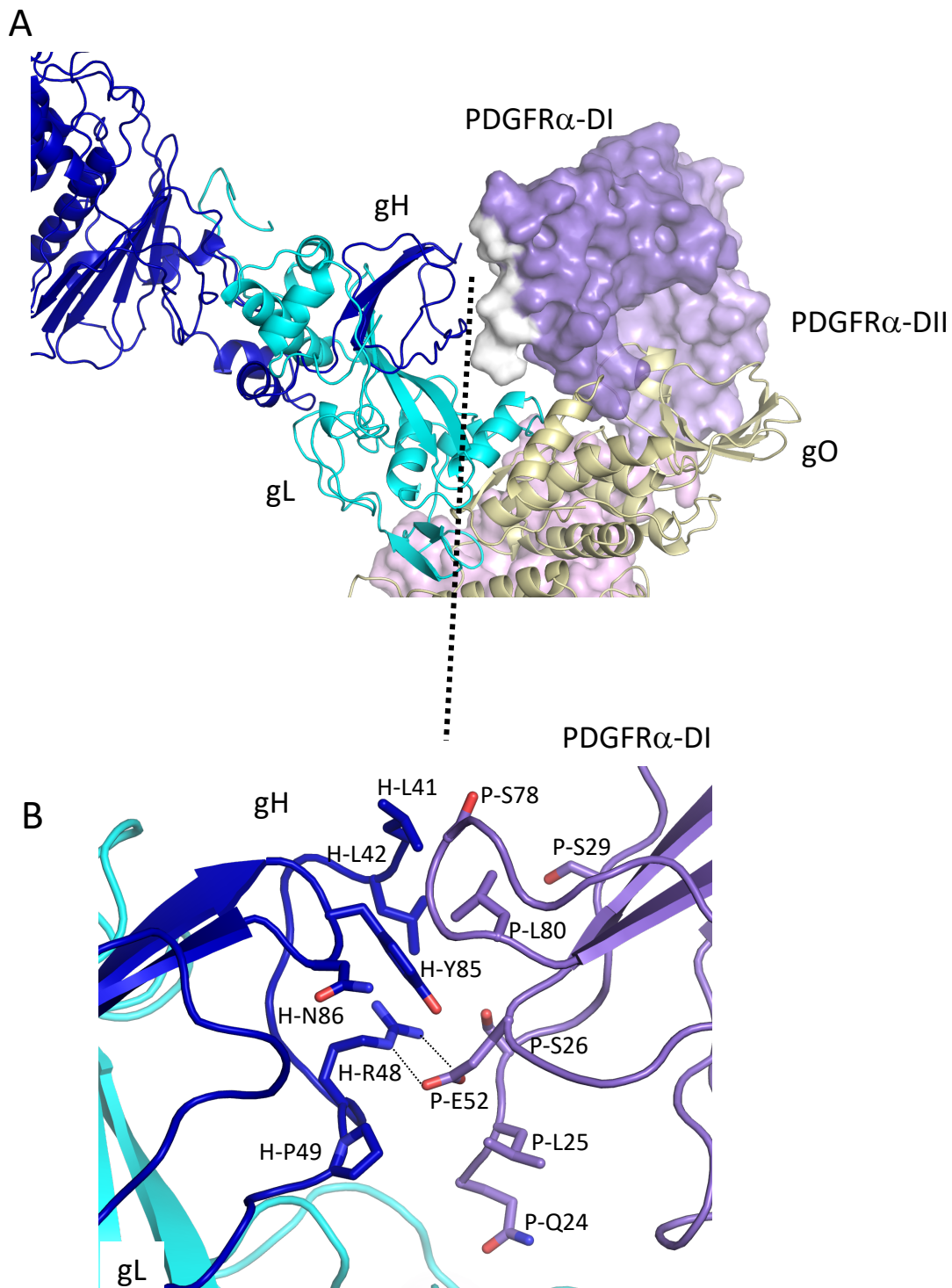

Supplementary Figure 7. PDGFR $\alpha$  interactions with gH

Supplement: FIG S7 [file mbio.02625-21-sf007.pdf]

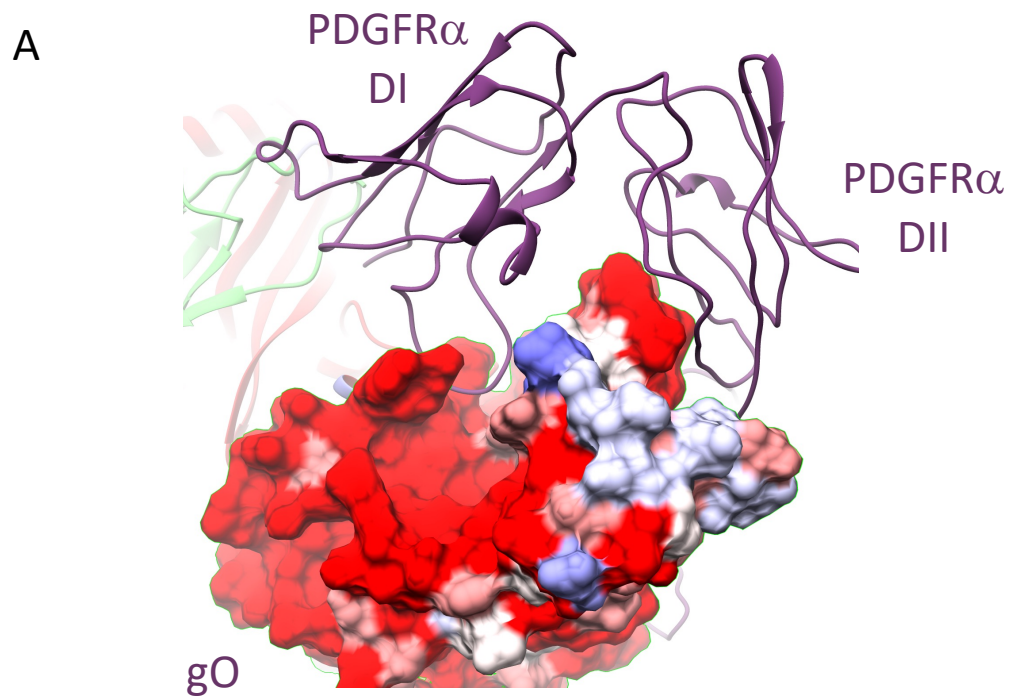

conserved variable

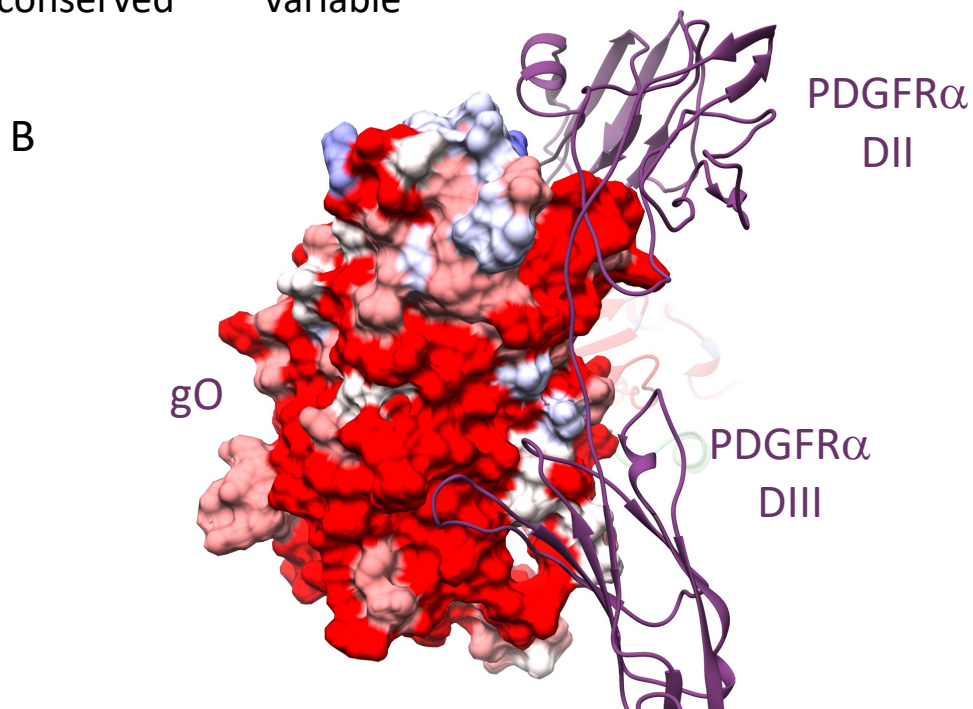

Supplementary Figure 8. Conservation of gO at PDGFR $\alpha$  interfaces

Supplement: FIG S8 [file mbio.02625-21-sf008.pdf]
