# Supplementary material for: Cryo-Electron Microscopy Structure and Interactions of the Human Cytomegalovirus gHgLgO Trimer with Platelet-Derived Growth Factor Receptor Alpha
Source: mBio. 2021 Oct 26;12(5):e02625-21. doi: 10.1128/mBio.02625-21 (PMC8546573; doi:10.1128/mBio.02625-21)
Supplement: TABLE S1 [file mbio.02625-21-st001.docx]

**Supplementary Table 1. Cryo-EM Data Collection and Model Refinement**

| **Data collection and processing** |  |
| --- | --- |
| Magnification | 130,000 |
| Voltage (kV) | 300 |
| Electron exposure (e^-^/Å^2^) | 75 |
| Defocus range (µm) | -1.4 to -2.5 |
| Pixel size (Å) | 1.06 |
| Symmetry imposed | C1 |
| Initial particle images | 4,190,429 |
| Final particle images | 345,997 |
| Map resolution (Å) | 3.43 |
| FSC threshold | 0.143 |
|  |  |
| **Refinement** | |
| Initial model | 5vob |
| Model composition | |
| Atoms | 23885 (hydrogens: 11877) |
| Proteins residues | 1497 |
| Map sharpening B factors (Å) | -122.6 |
| Protein B-factors (mean) | 101.05 |
| Bonds RMSD | |
| Length (Å) | 0.003 |
| Angles (°) | 0.531 |
|  | |
| **Validation** | |
| Molprobity score | 1.95 |
| Clashscore | 8.72 |
| Rotamer outliers (%) | 0 |
| Ramachandran plot | |
| Favored (%) | 92.19 |
| Allowed (%) | 7.81 |
| Outliners (%) | 0 |
| EMRinger score | 1.85 |
|  | |
| **Data Availability** | |
| EMDB | EMD-24369 |
| PDB | 7RAM |
