# Supplementary material for: Cryo-Electron Microscopy Structure and Interactions of the Human Cytomegalovirus gHgLgO Trimer with Platelet-Derived Growth Factor Receptor Alpha
Source: mBio. 2021 Oct 26;12(5):e02625-21. doi: 10.1128/mBio.02625-21 (PMC8546573; doi:10.1128/mBio.02625-21)
Supplement: FIG S3 [file mbio.02625-21-sf003.pdf]

1. AD169 gH
2. Merlin gH

```
                 10        20        30        40        50        60
1   1    MRPGLPPYLTVFTVYLLSHLPSQRYGADAASEALDPHAFHLLLNTYGRPIRFLRENTTQC   60
2   1    ......S..IILA.C.F...L.S....E.V..P..-K.......................   59
                 10        20        30         40        50

                 70        80        90       100       110       120
1   61   TYNSSLRNSTVVRENAISFNFFQSYNQYYVFHMPRCLFAGPLAEQFLNQVDLTETLERYQ  120
2   60   ............................................................  119
         60        70        80        90        100       110

                130       140       150       160       170       180
1   121  QRLNTYALVSKDLASYRSFSQQLKAQDSLGQQPTTVPPPIDLSIPHVWMPPQTTPHDWKG  180
2   120  ..............................E.........................G.TE  179
         120       130       140       150       160       170

                190       200       210       220       230       240
1   181  SHTTSGLHRPHFNQTCILFDGHDLLFSTVTPCLHQGFYLMDELRYVKITLTEDFFVVTVS  240
2   180  .......................................I....................  239
         180       190       200       210       220       230

                250       260       270       280       290       300
1   241  IDDDTPMLLIFGHLPRVLFKAPYQRDNFILRQTEKHELLVLVKKAQLNRHSYLKDSDFLD  300
2   240  ............................................D..........P....  299
         240       250       260       270       280       290

                310       320       330       340       350       360
1   301  AALDFNYLDLSALLRNSFHRYAVDVLKSGRCQMLDRRTVEMAFAYALALFAAARQEEAGT  360
2   300  ...........................................................A  359
         300       310       320       330       340       350

                370       380       390       400       410       420
1   361  EISIPRALDRQAALLQIQEFMITCLSQTPPRTTLLLYPTAVDLAKRALWTPDQITDITSL  420
2   360  QV.V...............................................N........  419
         360       370       380       390       400       410

                430       440       450       460       470       480
1   421  VRLVYILSKQNQQHLIPQWALRQIADFALQLHKTHLASFLSAFARQELYLMGSLVHSMLV  480
2   420  .............................K..............................  479
         420       430       440       450       460       470
```

```
1  481 HTTERREIFIVETGLCSLAELSHFTQLLAHPHHEYLSDLYTPCSSSGRRDHSLERLTRLF 540
2  480 ............................................................ 539

1  541 PDATVPATVPAALSILSTMQPSTLETFPDLFCLPLGESFSALTVSEHVSYVVTNQYLIKG 600
2  540 ..................................................I......... 599

1  601 ISYPVSTTVVGQSLIITQTDSQTKCELTRNMHTTHSITAALNISLENCAFCQSALLEYDD 660
2  600 ......................................V..................... 659

1  661 TQGVINIMYMHDSDDVLFALDPYNEVVVSSPRTHYLMLLKNGTVLEVTDVVVDATDSRLL 720
2  660 ............................................................ 719

1  721 MMSVYALSAIIGIYLLYRMLKTC 743
2  720 ....................... 742
```

Supplementary Figure 3. Sequence alignment of AD169 and Merlin gH
