# Supplementary material for: Cryo-Electron Microscopy Structure and Interactions of the Human Cytomegalovirus gHgLgO Trimer with Platelet-Derived Growth Factor Receptor Alpha
Source: mBio. 2021 Oct 26;12(5):e02625-21. doi: 10.1128/mBio.02625-21 (PMC8546573; doi:10.1128/mBio.02625-21)
Supplement: FIG S4 [file mbio.02625-21-sf004.pdf]

1. AD169 gL
2. Merlin gL

```
1   1    MCRRPDCGFSFSPGPVVLLWCCLLLPIVSSVAVSVAPTAAEKVPAECPELTRRCLLGEVF   60
2   1    ................I.............A.............................   60

1   61   QGDKYESWLRPLVNVTRRDGPLSQLIRYRPVTPEAANSVLLDDAFLDTLALLYNNPDQLR  120
2   61   E...............G.........................E.................  120

1   121  ALLTLLSSDTAPRWMTVMRGYSECGDGSPAVYTCVDDLCRGYDLTRLSYGRSIFTEHVLG  180
2   121  ............................................................  180

1   181  FELVPPSLFNVVVAIRNEATRTNRAVRLPVSTAAAPEGITLFYGLYNAVKEFCLRHQLDP  240
2   181  ............................................................  240

1   241  PLLRHLDKYYAGLPPELKQTRVNLPAHSRYGPQAVDAR  278
2   241  ......................................  278
```

Supplementary Figure 4. Sequence alignment of AD169 and Merlin gL
