# Supplementary material for: Cryo-Electron Microscopy Structure and Interactions of the Human Cytomegalovirus gHgLgO Trimer with Platelet-Derived Growth Factor Receptor Alpha
Source: mBio. 2021 Oct 26;12(5):e02625-21. doi: 10.1128/mBio.02625-21 (PMC8546573; doi:10.1128/mBio.02625-21)
Supplement: FIG S5 [file mbio.02625-21-sf005.pdf]

1. Merlin gO (gO5 genotype)
2. VR1814 gO (gO1c genotype)
3. TR gO (gO1b genotype)

```
1  MGKKEMIMVKGIPKIMLLISITFLLLSLINCNVLVNSRGTRRSWPYTVLSYRGKEILKKQKEDILKRLMS   70
2  MGRKEDM--RSISKLFFIISLTVLLFSIINCKVV---RPPGRYWLGTVLSTIGKQKLDKFKLEILKQL--   63
3  MGRKGEM--RGVFNLFFLMSLTFLLFSFINCRAAVR-LSVGRYWSGKVLSTIGKQRLDKFKLEILKQL--   65

1  TSSDGYRFLMYPSQQKFHAIVISMDKFPQDYILAGPIRNDSITHMWFDFYSTQLRKPAKYVYSEYNHTAH  140
2  -EREPYTKYFNMTRQHVKNLTMNMTQFPQYYILAGPIRNDSITYLWFDFYSTQLRKPAKYVYSQYNHTAK  132
3  -EKDIYTKYFNMTRQHIKNLTMNMTEFPRYYILAGPIQNNSVTYLWFDFYSTQLRKPAKYVFSEYNHTAK  134

1  KITLRPPPCGTVPSMNCLSEMLNVSKRNDTGEKGCGNFTTFNPMFFNVPRWNTKLYIGSNKVNVDSQTIY  210
2  TITFRPPSCGTVPSMTCLSEMLNVSKRNDTGEQGCGNFTTFNPMFFNVPRWNTKLYVGPTKVNVDSQTIY  202
3  TITFRPPSCGTVPSMTCLSEMLNVSKRNDTGEQGCGNFTTFNPMFFNVPRWNTKLYVGSKKVNVDSQTIY  204

1  FLGLTALLLRYAQRNCTRSFYLVNAMSRNLFRVPKYINGTKLKNTMRKLKRKQALVKEQPQKKNKKSQST  280
2  FLGLTALLLRYAQRNCTHSFYLVNAMSRNLFRVPKYINGTKLKNTMRKLKRKQAPVKEQLEKKTKKSQST  272
3  FLGLTALLLRYAQRNCTHSFYLVNAMSRNLFRVPKYINGTKLKNTMRKLKRKQAPVKEQSEKKSKKSQST  274

1  TTPYLSYTTSTAFNVTTNVTYSATAAVTRVATSTTGYRPDSNFMKSIMATQLRDLATWVYTTLRYRNEPF  350
2  TTPYFSYTTSTALNVTTNATYRVTTSAKRIPTSTIAYRPDSSFMKSIMATQLRDLATWVYTTLRYRNEPF  342
3  TTPYSPYTTSTALNVTTNATYSVTTTARRVSTSTIAYRPDSSFMKSIMTTQLRDLATWVYTTLRYRQNPF  344

1  CKPDRNRTAVSEFMKNTHVLIRNETPYTIYGTLDMSSLYYNETMSVENETASDNNETTPTSPSTRFQRTF  420
2  CKPDRNRTAVSEFMKNTHVLIRNETPYTIYGTLDMSSLYYNETMSVENETASDNNETTPTSPSTRFQKTF  412
3  CESSRNRTAVSEFMKNTHVLIRNETPYTIYGTLDMSSLYYNETMFVENKTAS---ETTPTSPSTGFQRTF  411

1  IDPLWDYLDSLLFLDKIRNFSLQLPAYGNLTPPEHRRAANLSTLNSLWWWSQ  472
2  IDPLWDYLDSLLFLDKIRNFSLQLPAYGNLTPPEHRRAVNLSTLNSLWWWLQ  464
3  IDPLWDYLDSLLFLDEIRNFSLQSPTYGNLTPPEHRRAVNLSTLNSLWWWLQ  463
```

Supplementary Figure 5. Sequence alignment of Merlin, TR and VR1814 gO sequences
