# Supplementary material for: Cryo-Electron Microscopy Structure and Interactions of the Human Cytomegalovirus gHgLgO Trimer with Platelet-Derived Growth Factor Receptor Alpha
Source: mBio. 2021 Oct 26;12(5):e02625-21. doi: 10.1128/mBio.02625-21 (PMC8546573; doi:10.1128/mBio.02625-21)
Supplement: TEXT S1 [file mbio.02625-21-s0001.docx]

**Sequences used for gH, gL and gO variability analysis**

**gH sequences (UniprotKB accession)**

A8T7F0, Q69160, A0A2H4GG95, Q68571, A1IKL5, V9PVB8, A1IKM1, A0A2I2MML4, A0A2H4GLJ3, A0A0R5E2N6, A0A0G2UDD2, A1IKL6, A0A2H4GPX9, A0A0G2TDK6, A0A6M3EUN0, A0A1L3IXF8, Q69161, X4YGS2, A0A0G2T8B4, A0A126N900, E7DVL5, A1IKL8, A1IKL4, A0A0G2TYY1, A1IKM0, A0A0G2TEE1, A0A0R5E7E7, A0A0G2TKV9, C8BKM2, A0A0G2UCF9, A0A0G2TNQ6, D2K4W8, Q69155, A0A0G2UJE4, Q69158, R4S5R4, A0A0A7CFY1, Q69162, Q6RXF7, V9LPM1, Q69157, D2K5H1, A0A1L3IYG2, A0A0G2TPJ9, A0A0G2TK29, A0A2H4GFB0, A0A6C2E2A3, A0A126NBV8, A0A162DXL8, A0A0A0PIH4, A0A6M3ETD3, A0A140II56, V9LQY5, A0A0G2TKS8, A1IKL7, C8CFB4, A0A0G2UK76, A1IKL9, A0A0G2ULH8, A0A455R9C8, V9PU18, A0A0G2TW06, A0A1L3IXZ0, A0A0G2U208, V9LS46, A0A0R5EBE5, A0A2H4GKK4, A0A126NHY2, A0A0G2TQI3, A0A0G2T6I9, A0A0G2TSI3, A0A0G2TM81, A0A6C2E5M5, A0A0R5EFW1, A1IKM2, A0A140D1A5, A0A159B2D1, A0A0G2U3D3, A0A0R5EE19, A0A2H4GP09, A0A0G2TMQ5, Q89713, V9LMR1, Q69156, F5H9T3, C8CPG6, A0A0G2TFY4, C8BL19, D2K4J1, A0A0G2TIM2, C5MKS1, A0A0G2U7U1, C8BLF2, Q69159

**gL sequences (UniprotKB accession)**

Q68672, F5HCH8, P16832, Q68670, Q68669, Q68667, Q68673, Q68668, Q68671, Q68666, D5LXA3, B9VXR5, B8YED2, A0A6C2E462, C8CFW1, A0A2H4GEX3, A0A126NDV5, A0A0G2TIJ2, A0A0R5E540, A0A0G2T6I1, A0A6M3EQ94, A0A6C2E2E6, A0A0G2U150, A0A0G2UJF0, A0A6M3EUH7, Q8JP82, A0A0G2TJV9, A0A0R5E2R3, A0A0G2U4T7, A0A0R5EBI0, Q8JP78, A0A0G2TB74, A0A6C2E3J8, D3YS28, A0A0G2U8B5, A0A2H4GRP0, X4YYT7, A0A126NDD1, Q8JP79, A0A0G2TE97, A0A0A7CH53, A0A162DY86, A0A5S9EFP9, A0A0G2TPA2, A0A0G2U3E9, A0A159B2A0, Q8JP81, A0A0G2U9D4, A0A6C2E767, A0A159B0V2, V9LLZ9, A0A1I9XFF3, A8T7J6, V9PVC6, Q8JJD7, Q8JP83, V9LPD9, R4SHE6, A0A1I9XCT8, C8CFE6, Q8JJF0, Q71DP4, A0A0A0PKG9, A0A0G2U3K4, Q8JP80, D2K5K3, A0A0G2TIH5, A0A0G2TCT2, Q8JJH5, C8BKQ4, Q8JJK9, A0A0G2U1H8, Q71DN9

**gO_sequences (UniprotKB accession)**

F5HGP1, Q8BCV1, A0A2H4GL51, B2L067, B2L066, A0A455R9W9, B2L065, A0A0G2UF28, C8BLF0, A0A0A0PKJ9, P16750, Q8BCU8, A0A0G2TGN6, Q8AZ36, B2L072, Q8BCU2, D2IGU0, D2IGF0, A0A2H4GJM5, A0A126N8J6, Q8AZ28, B2L071, Q71DI2, B8YE99, A0A0G2U6C7, B2L070, A0A2H4GLV2, A0A0G2TZM5, A0A0R5E4V8, Q8AYW0, A0A162DRU8, A0A0G2TNN0, Q8BCU9, D2IGJ5, D2IGD5, D2IGI0, D2K5T5, A0A0G2TTG2, D2IGN5, A0A0G2TZ21, Q8BCT8, D2IGT5, Q8BCU3, A0A126NDR8, A0A0G2U3V7, A0A159B4K2, A0A159B2M8, A0A0G2TC28, A0A0G2TD53, Q8AYZ5, V9LM06, A0A126NBX1, Q8BCU4, Q8AYV5, A0A162DYP2, Q8BCU1, A0A0G2U655, A0A140D1A3, A0A1L3IXE6, V9PUM4, D2IGL0, A0A0G2TCN3, Q8BCT9, A0A0G2T792, Q8AZ39, Q8BCV2, Q8AZ32, A0A0G2U2E6, A0A2H4GII8, A0A0G2U4B4, A0A0G2UI88, D2IGR0, A0A159B107, A0A0G2U6Q1, A0A0G2TYL5, B2L063, A0A159B5U4, A0A0G2T9C4, D2IGE5, Q8AYZ3, A0A0R5EB21, C8CFB2, D2IGD0, Q8BCU5, Q8BCU6, Q8BCU0, A0A1I9XDS5, D2IGH0, Q8BCV0, B9VXN2, X4YJ27, A0A0G2UIN5, A0A2S0DCH1, A0A1I9XC16, C8CPG4, Q8BCT7
